# Nemo-like kinase limits T cell activation while preserving anti-tumor responses

**DOI:** 10.1016/j.isci.2026.118070

**Published:** 2026-11-02

**Authors:** Sebastian Peer, Jiří Koutník, Nikolaus Thuille, Kerstin Siegmund, Victoria Klepsch, Maria Pfeiffer, Dominik Humer, Patrick Bantsich, William J. Olson, Karin Albrecht-Schgoer, Gottfried Baier, Thomas Gruber

**Affiliations:** 1 Institute of Cell Genetics, Department for Genetics, Medical University of Innsbruck, 6020 Innsbruck, Austria; 2 University Hospital for Internal Medicine V (Haematology and Oncology, Cytometry Unit), Medical University of Innsbruck, 6020 Innsbruck, Austria; 3 Former Member of the Institute of Cell Genetics, Department for Genetics, Medical University of Innsbruck, 6020 Innsbruck, Austria; 4 German Cancer Research Center (DKFZ), 69120 Heidelberg, Germany; 5 Institute for Biomedical Aging Research, University of Innsbruck, 6020 Innsbruck, Austria

**Keywords:** nemo-like kinase, T cell activation, T cell dysfunction, anti-tumor immunity

## Abstract

T cell dysfunction limits the efficacy of cancer immunity, yet the molecular checkpoints that preserve T cell function within the tumor microenvironment are not fully understood. In this study, we identify a previously unrecognized, T cell-intrinsic role for nemo-like kinase (NLK) as a critical mediator of sustained anti-tumor responses. In our experiments, NLK emerged as a dual-function modulator: although NLK deficiency in CD4^+^ and CD8^+^ T cells increases proliferation and cytokine production *in vitro* via enhanced NFAT and AP-1 activation, its loss *in vivo* paradoxically impairs tumor control. In murine tumor models, NLK-deficient tumor-infiltrating T cells exhibit blunted effector activity resulting in accelerated tumor progression. Conversely, human chimeric antigen receptor (CAR) T cells that overexpress NLK demonstrate superior killing capacity toward target tumor cells. These findings establish NLK as an essential early brake on T cell activation while protecting against their chronic dysfunction, and reveal a molecular pathway that sustains anti-tumor immunity.

## Highlights

• NLK uncouples acute T cell activation from sustained anti-tumor immunity
• NLK loss enhances T cell activation *in vitro* but impairs tumor control *in vivo*
• NLK-deficient tumor-infiltrating T cells show reduced effector activity
• NLK overexpression enhances the killing capacity of human CAR T cells

## Graphical abstract

**Figure:**
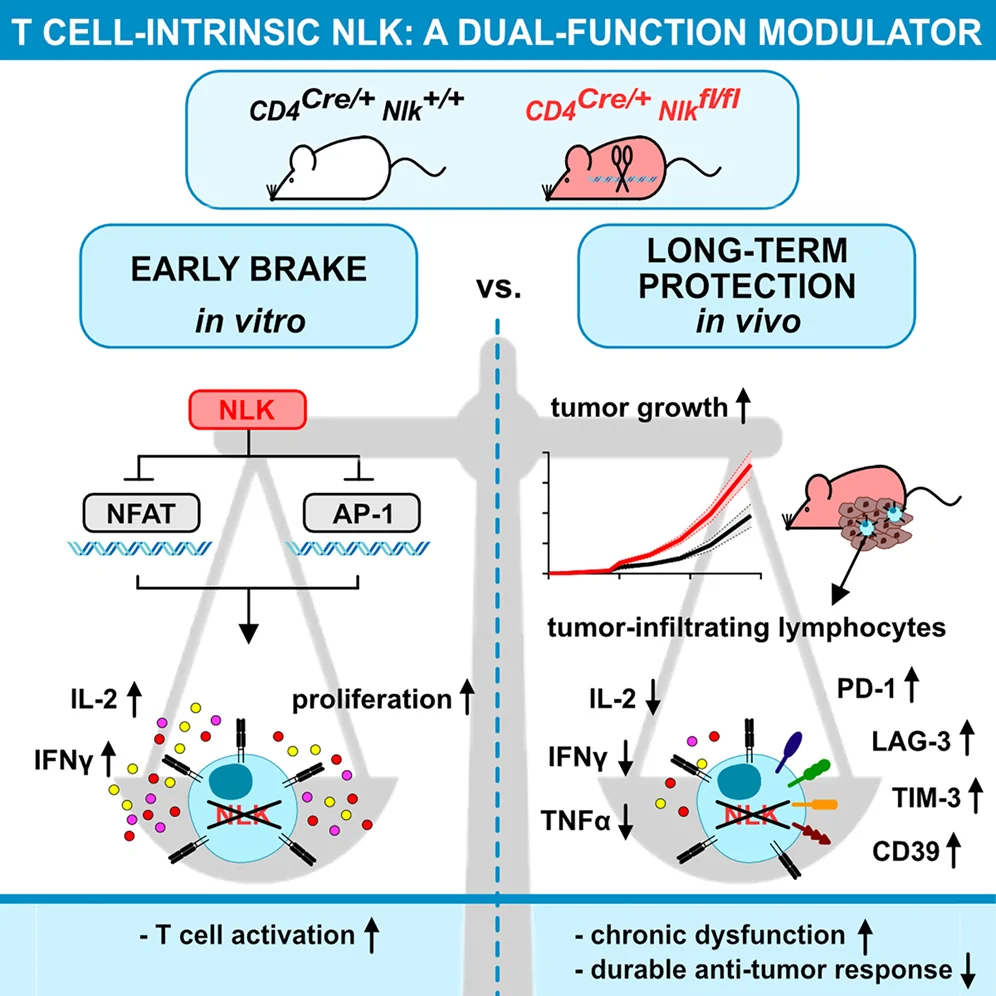

## Teaser

Natural sciences; Biological sciences; Cancer systems biology; Cancer

## Introduction

Over the last years, the so-called “cancer immunosurveillance hypothesis”, initially formalized by Macfarlane Burnet and Lewis Thomas,^1^^,^^2^^,^^3^^,^^4^ has been impressively confirmed, with many of the underlying mechanisms being revealed. It has been proven that the immune system could indeed recognize and eliminate cancer cells. Especially, the CD8^+^ T cell compartment turned out to be a crucial effector of antitumor immunity.

However, with growing knowledge about CD8^+^ T cell fates, it became evident that—besides differentiation into effector and memory cells—T cells can also get hyporesponsive. This state of T cell dysfunction is characterized by loss of effector functions and high expression of inhibitory receptors (e.g., PD-1, TIM-3, and LAG-3). It was first described in the setting of chronic viral infection^5^^,^^6^^,^^7^^,^^8^ and later extended to tumor immunology.^9^ This is clearly clinically relevant: during the last decade, T cell-based cancer immunotherapy has shown encouraging success; however, all of these therapies are restricted by fading T cell responses due to the development of T cell dysfunction or insufficient persistence.^10^ Therefore, there is an urgent need to improve our understanding of the underlying mechanisms that lead to the development of poorly functional T cells.

Nemo-like kinase (NLK) is a serine-threonine kinase whose amino acid sequence is 54.5% similar and 41.7% identical to that of mitogen-activated protein kinase-1 (in mice).^11^ *Nlk* knockout mice on a C57BL/6 background die in the third trimester of pregnancy, whereas those on a 129/Sv background survive up to 4–6 weeks after birth,^12^ indicating a role in early development. It was shown that NLK plays crucial roles in the regulation of diverse signaling pathways, including the Wnt/β-catenin and Notch pathways, and is involved in embryonic patterning, nervous system development, and cancer cell proliferation.^13^ Regarding the functions of NLK in T lymphocytes, Fleskens et al. reported that NLK sustains Foxp3 transcriptional activity, thereby maintaining the suppressive function of regulatory T cells.^14^ In the work of Daams et al., deletion of NLK leads to a reduction of single-positive CD8^+^ T cells in the thymus due to increased cell death in this population.^15^ However, there is lack of knowledge about functions of NLK in peripheral mature effector T cells.

During our screen for T cell-relevant proteins and signaling pathways, we discovered NLK as a kinase that plays a crucial role in preventing T cell dysfunction in the context of anti-tumor immunity. We found that mice with a T cell-specific deletion of *Nlk* were unable to control tumor growth sufficiently. Although NLK-deficient T cells were initially hyperreactive, tumor-infiltrating T cells lacking NLK exhibited reduced effector functions and increased inhibitory receptor expression. These data were largely confirmed *in vitro* by human T cells that overexpressed NLK. Therefore, our data suggest that NLK acts as a negative regulator of T cell activation but prevents T cell dysfunction in the context of cancer immunity, thereby enhancing T cell effector functions against tumor cells.

## Results

### Generation of tissue-specific *Nlk* knockout mice

To study the functions of NLK in mature T cells, we generated tissue-specific *Nlk* knockout mice because whole-body *Nlk* knockout mice have a very short lifespan.^16^ We crossed mice possessing *loxP* sites flanking exon 2 of the *Nlk* gene with mice expressing the Cre recombinase under the control of the CD4 promoter (Figure S1A). This resulted in mice with an *Nlk* deletion that is largely restricted to T cells (hereafter referred to as NLK^fl/fl^). Diminished NLK expression was confirmed by Western blot and RT-PCR analyses which compared the amounts of protein and mRNA in NLK^fl/fl^ and *Nlk*^+/+^ CD4-Cre^+^ (hereafter referred to as NLK^+/+^) T cells (Figures S1B and S1C).

### Characterization of T cell-specific *Nlk* knockout mice

When we monitored the weight of the mice up to 70 days of age, we found no difference between the genotypes (Figure S2A). Also at one year of age, spleen and mouse weight did not significantly differ between NLK^fl/fl^ and NLK^+/+^ genotypes (Figure S2B). The thymic development of T cells was not affected by the loss of NLK, as evidenced by unchanged percentages and numbers of CD4 and CD8 double-positive and single-positive thymocytes as well as regulatory T cells (T_regs_) of 12-week-old mice (Figure S3). In particular, and in contrast to a recent study performed with Lck-Cre *Nlk*^flfl^ mice,^15^ we found no decrease in the percentage and absolute number of NLK-deficient CD8 single-positive cells in the thymus (percentage: NLK^+/+^ 1.65 ± 0.06% vs. NLK^fl/fl^ 2.06 ± 0.16%; *p* = 0.049, *n* = 6; absolute: NLK^+/+^ 1.68 ± 0.15 × 10^6^ vs. NLK^fl/fl^ 2.4 ± 0.34 × 10^6^; *p* = 0.084, *n* = 6).

Examination of T cell subsets in the peripheral lymphoid organs revealed that the distribution and total numbers of these subsets were comparable between NLK^fl/fl^ mice and their controls, except for the CD8^+^ effector/memory compartment, which was reproducibly smaller in 8–12-week-old NLK^fl/fl^ animals (NLK^+/+^ 6.9% ± 1.1% of all CD8^+^ vs. NLK^fl/fl^ 3.9% ± 0.4%, *p* = 0.035, *n* = 6) (Figure S4A). This reduction was still observable in the spleens of 28 weeks old animals (NLK^+/+^ 15.1% ± 1.2% of all CD8^+^ vs. 10.5% ± 1.2%, *p* = 0.032) (Figure S4B).

Notably, the frequency of CD4^+^CD25^+^Foxp3^+^ T_regs_ in peripheral lymphoid organs was not altered in 12- or 28-week-old NLK^fl/fl^ mice (Figure S4). Furthermore, *in vitro* T_reg_ differentiation of NLK^fl/fl^ from naive CD4^+^ T cells (as determined by Foxp3 expression) using IL-2 and TGF-β as skewing cytokines was normal (Figure S5A). However, NLK-deficiency moderately impaired the ability of regulatory T cells to suppress effector T cells *in vitro* (Figure S5B).

Taken together, these results demonstrate that the T cell-specific loss of NLK does not affect body or spleen weight, thymocyte development and the distribution of peripheral T cell subsets, apart from a relative decrease of CD8^+^ effector/memory T cells. Of note, NLK-deficiency diminishes the suppressive capacity of CD4^+^ T_regs_ toward effector T cells.

### NLK-deficient T cells are hyperreactive *in vitro*

As shown in Figure S1B, we found that *Nlk* expression is inducible upon T cell activation, suggesting a role in a feedback loop for the control of adaptive immune responses. To investigate this further, we activated CD4^+^ and CD8^+^ T cells *in vitro* with anti-CD3 and anti-CD28 antibodies and observed that NLK-deficient T cells proliferated at a higher rate than their wild-type counterparts as measured by ^3^H-thymidine incorporation (Figure 1A). This result was confirmed by tracking cell divisions using the fluorescent dye carboxyfluorescein succinimidyl ester (CFSE) (Figure 1B). In addition, we determined cytokine concentrations in the supernatants of the cultured cells and found that in the absence of NLK, activated CD4^+^ and CD8^+^ T cells produced more IL-2 and IFN-γ, respectively (Figure 1C). This enhanced IFN-γ expression by CD8^+^ T cells was observed also at the mRNA level as early as 6 h after stimulation (Figure 1D). Taken together, these results show that NLK-deficient T cells are hyperreactive in the first days after stimulation, implicating NLK as a negative regulator of the early events leading to T cell proliferation and cytokine production.

**Figure 1:**
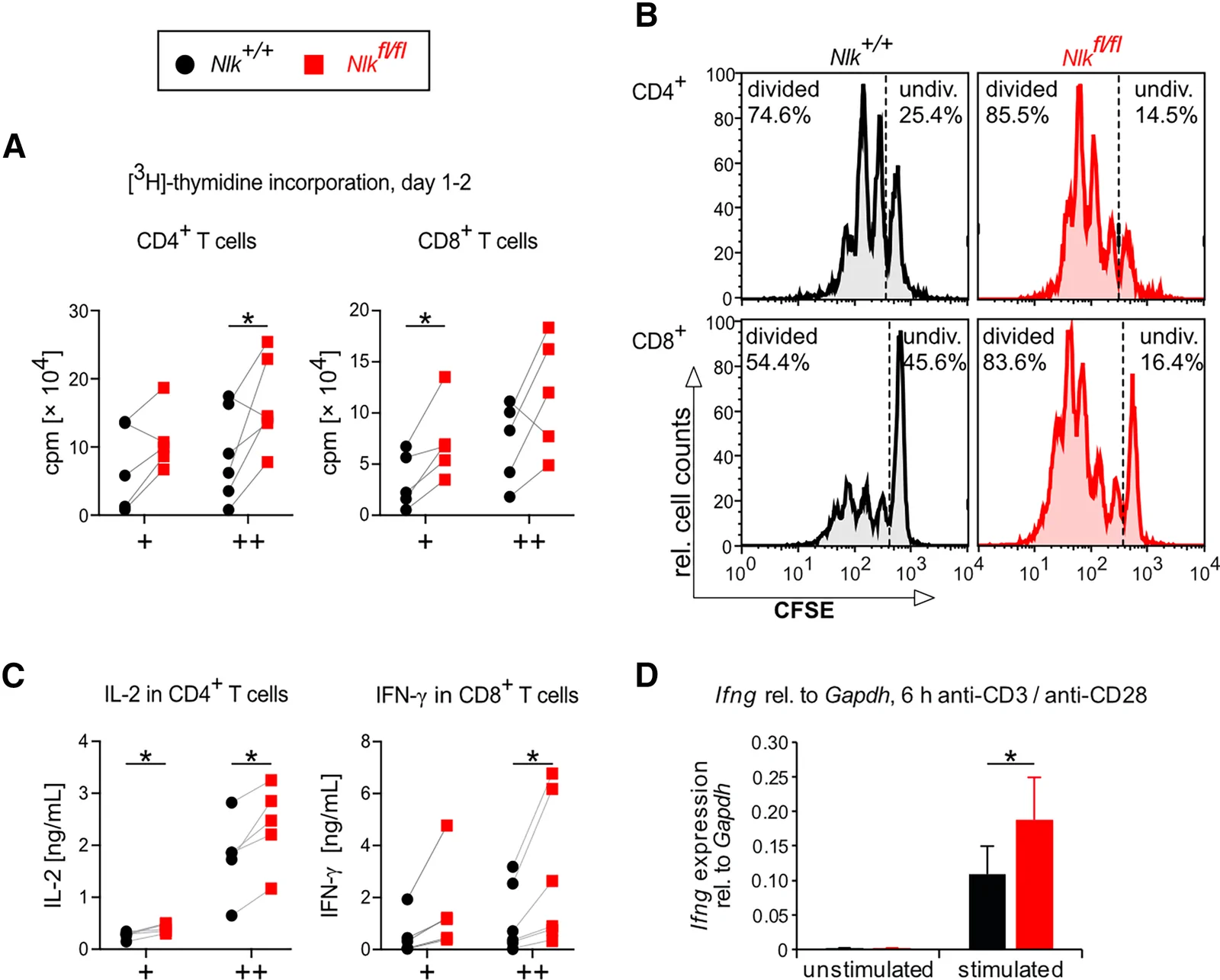
Proliferation and cytokine production of CD4^+^ and CD8^+^ T cells (A and C) Wild-type and NLK-deficient T cells were stimulated with 2 μg/mL plate-bound anti-CD3 with (++) or without (+) 1 μg/mL soluble anti-CD28. (A) Proliferation was measured via [3H]-thymidine uptake. (B) Wild-type and NLK-deficient lymph node and spleen cells were stimulated with 2 μg/mL plate-bound anti-CD3 and 1 μg/mL soluble anti-CD28. Proliferation was analyzed by flow cytometry based on CFSE dilution after three days. (C) IL-2 secretion by CD4^+^ cells and IFN-γ secretion by CD8^+^ cells was determined with Bio-Plex after one day of stimulation. (D) Wild-type and NLK-deficient CD8^+^ T cells were stimulated with 2 μg/mL plate-bound anti-CD3 and 1 μg/mL soluble anti-CD28 for 6 h or left unstimulated. IFN-γ expression was determined by RT-qPCR and depicted as relative amount to GAPDH; *n* = 5. Data are represented as mean ± SEM. Paired *t* test; ∗*p* < 0.05.

T cell activation is accompanied by translocation of the crucial transcription factors NFAT, AP-1, and NF-κB into the nucleus where they control the expression of various genes. When we compared the binding of NFAT to its consensus DNA sequence, we found that NFAT activation was strongly enhanced in NLK-deficient T cells, as reflected by increased nuclear translocation and DNA binding (Figures 2A–2C). Additionally, the binding of AP-1 to its DNA consensus sequence was increased in NLK^fl/fl^ T cells (Figure 2D). In contrast, the nuclear translocation of p50, an important member of the NF-κB family, was unaffected by the loss of NLK (not shown).

**Figure 2:**
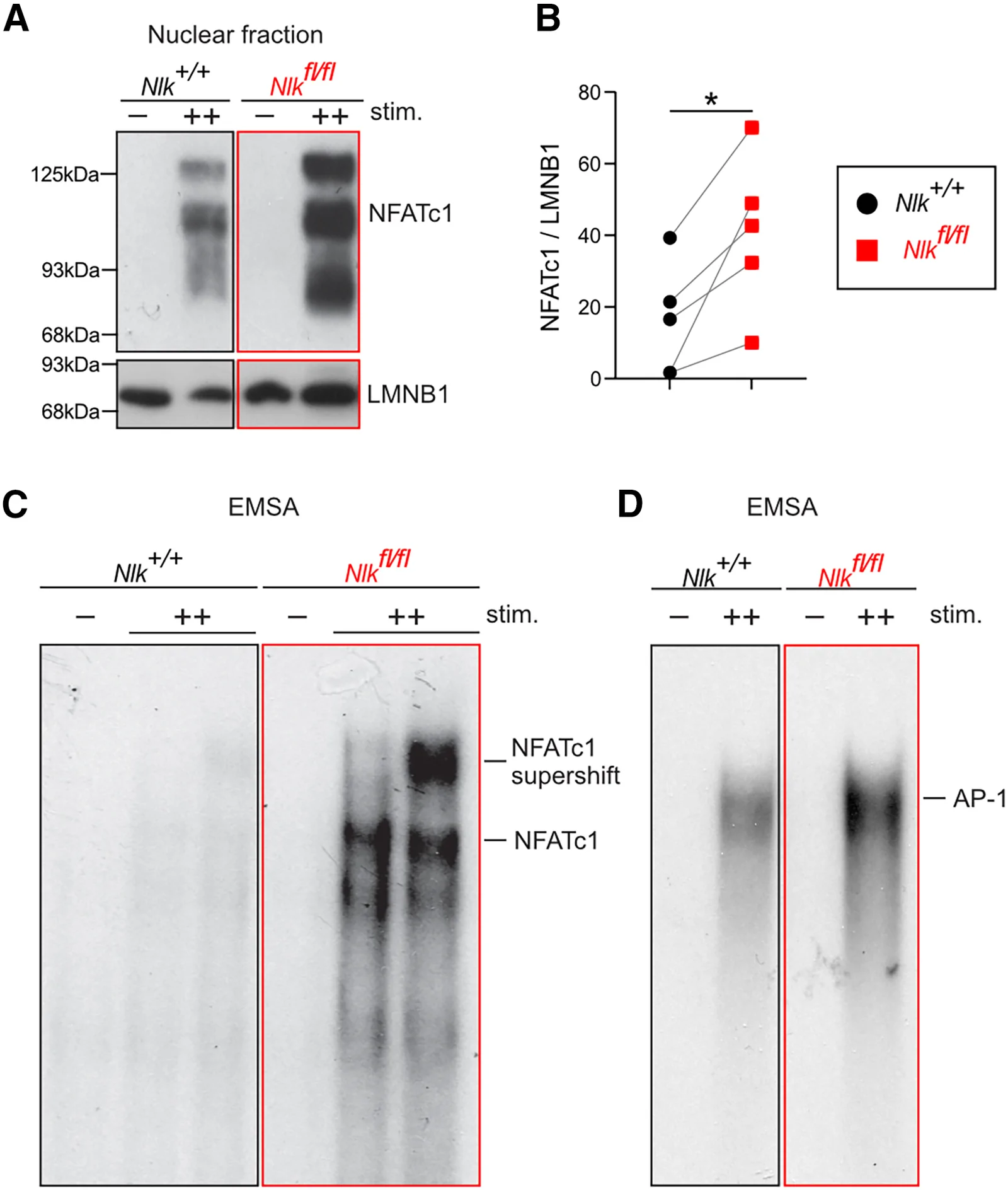
NFATc1 and AP-1 activation Wild-type and NLK-deficient CD8^+^ T cells were left unstimulated (−) or stimulated with 2 μg/mL plate-bound anti-CD3 and 1 μg/mL soluble anti-CD28 for 22 h (++). (A) Nuclear extracts were analyzed on immunoblots to measure nuclear entry of NFATc1. Lamin B1 (LMNB1) was used as a loading control. One representative experiment out of five is shown. (B) Densitometric analysis of five independent immunoblots. Nuclear NFATc1 amounts are relative to loading control. (C) Equal amounts of nuclear extracts were analyzed by electrophoretic mobility shift assay to assess DNA binding of NFATc1. For supershift, 1 μg anti-NFATc1 was added to the binding reaction. One representative experiment out of three is shown. (D) Nuclear extracts were analyzed by electrophoretic mobility shift assay to assess DNA binding of AP-1. One experiment out of two with similar results is shown. Paired *t* test; ∗*p* < 0.05.

These results suggest that NLK negatively regulates early NFAT and AP-1 activation and consequently proliferation and cytokine expression of T cells.

### NLK-deficient T cells are hyporeactive in antitumor immunity

CD8^+^ T cells are considered the crucial component of adaptive antitumor immunity due to their direct cytotoxic effector function. Given the hyperactive phenotype of NLK-deficient T cells *in vitro*, we hypothesized that these cells could control tumor growth better than their wild-type counterparts. This would open the possibility of testing NLK as a pharmacological target to enhance T cell functions in cancer immunotherapy. Remarkably, however, when we injected 250,000 MC38 colon carcinoma cells subcutaneously into NLK^fl/fl^ and NLK^+/+^ mice, we observed the opposite: In T cell-specific *Nlk* knockout mice, tumors grew faster than in the control animals (Figure 3A), and tumor weight on day 21 after injection was three times higher in NLK^fl/fl^ compared to NLK^+/+^ mice (Figure 3B). This result was confirmed by using a second ectopic tumor model, in which the transformed lung epithelial cell line TC-1 was injected subcutaneously (Figure 3C).

**Figure 3:**
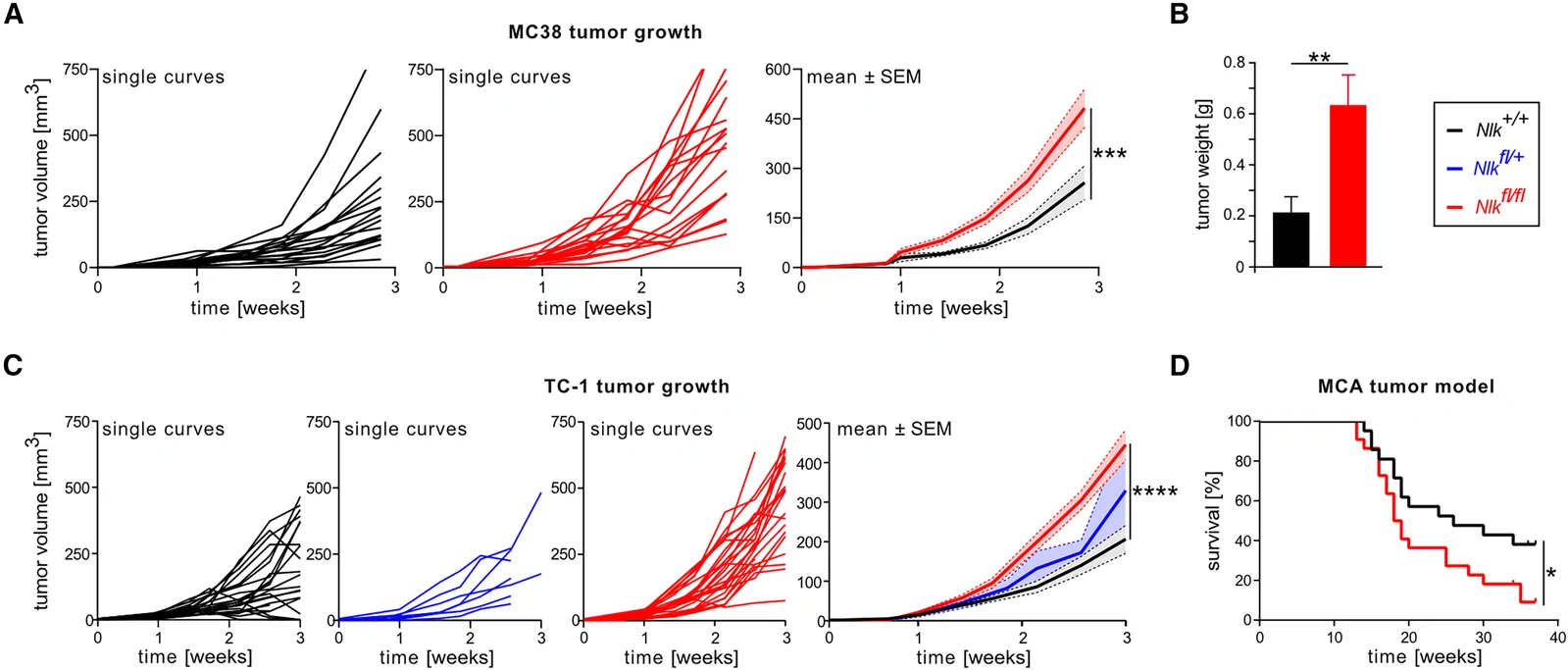
T cell-specific deletion of NLK impairs tumor control *in vivo* (A) 250,000 MC38 colon carcinoma cells were injected subcutaneously and tumor volume was monitored. Mean and SEM are shown, *n* = 17–18. (B) Weight of MC38 tumors 21 days after injection. Mean and SEM are shown, *n* = 17–18. (C) 150,000 transformed TC-1 lung epithelial cells were injected subcutaneously and tumor volume was monitored. Mean and SEM are shown, *n* = 25 (NLK^+/+^and NLK^fl/fl^) and *n* = 7 (NLK^fl/+^). (D) Survival of mice after subcutaneous injection of 0.2 mg 3-methylcholanthrene (MCA), *n* = 21–22. Two-way ANOVA (A and C), unpaired *t* test (B), and Cox regression (D); ∗*p* < 0.05, ∗∗*p* < 0.01, ∗∗∗*p* < 0.001, ∗∗∗∗*p* < 0.0001.

Furthermore, in an autochthonous tumor model, NLK deficiency was significantly associated with tumor progression (HR = 2.12, 95% CI: 1.03–4.37, *p* = 0.042), and the survival time of NLK^fl/fl^ mice was significantly shortened compared to wild-type controls (median survival 18.5 vs. 26 weeks) (Figure 3D). In this model, the carcinogen methylcholanthrene is applied subcutaneously and the tumors develop spontaneously. Since NLK loss is largely restricted to T cells in our model, these results indicate that NLK-deficient T cells are less capable of controlling tumor growth than their wild-type counterparts.

When we examined the tumor-infiltrating lymphocytes (TILs) of MC38 tumors, we found no difference in the distribution of CD4^+^ or CD8^+^ T cells, T_regs_, NKT (natural killer T) cells, and B cells. Also, the percentage of total leukocytes (CD45^+^) in the tumors was not altered between the genotypes. However, in NLK^fl/fl^ mice, there was a small shift from central to effector memory cells within the CD4^+^ compartment and a small but significant reduction in the percentage of tumor-infiltrating natural killer (NK) cells (Figures 4A and 4B and Table S1).

**Figure 4:**
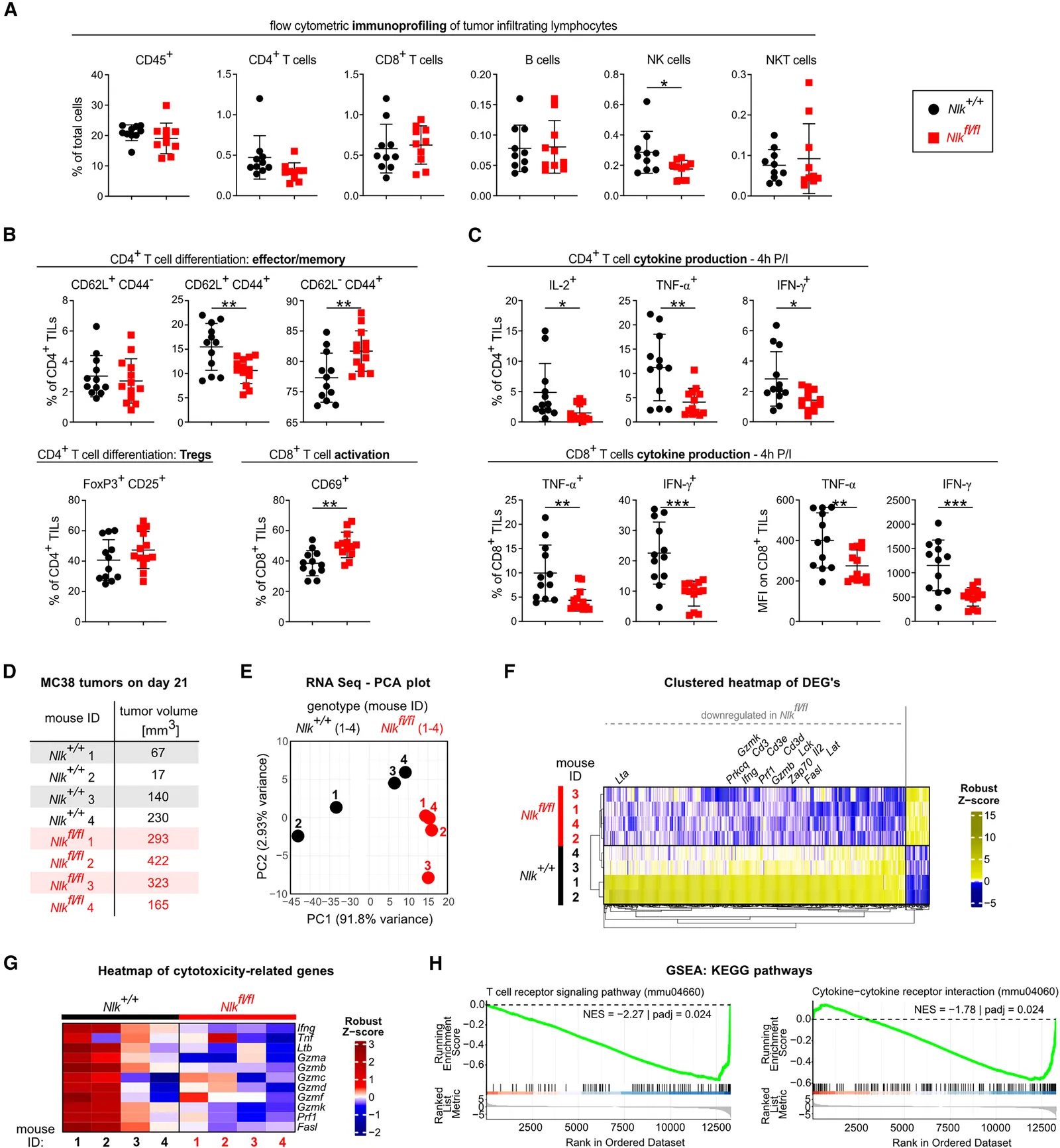
Flow cytometric and RNA-seq analyses of MC38 tumor-infiltrating lymphocytes Flow cytometric analyses were performed three weeks after MC38 injection. (A) Percentage of immune cells (CD45^+^), T cells (CD4^+^ or CD8^+^), B cells (CD19^+^), NK cells (CD3^−^NK1.1^+^), and NKT cells (CD3^+^NK1.1^+^). (B) Distribution of CD4^+^ naive (CD62L^+^CD44^−^), central memory (CD62L^+^CD44^+^), effector memory (CD62L^−^CD44^+^), regulatory (Foxp3^+^CD25^+^), and CD8^+^CD69^+^ T cells. (C) Intracellular staining of cytokines in CD4^+^ and CD8^+^ T cells after stimulation with phorbol-12,13-dibutyrate and ionomycin (P/I) for 4 h. (D) Volumes of the MC38 tumors used in the RNA-seq experiments. (E–H) RNA-seq analyses of CD8^+^ TILs were performed on normalized gene expression profiles obtained from RNA sequencing of tumor-infiltrating CD8^+^ T cells isolated from four NLK^+/+^ and four NLK^fl/fl^ mice three weeks after MC38 injection. (E) Principal component analysis (PCA). The plot shows the separation of samples based on DESeq2-normalized overall transcriptomic profiles. (F) Clustered heatmap of differentially expressed genes (DEGs), defined as log2 fold change ≥1 and *p* adj ≤0.05. Due to space limitations, some genes that are located below each other in the heatmap are written next to each other. (G) Heatmap of cytotoxicity-related genes. (F and G) Robust *Z* scores are based on median and interquartile range. (H) Gene set enrichment analysis (GSEA) plots for the indicated KEGG pathways. Normalized enrichment score (NES) and adjusted *p* value (*p* adj) are shown. (A–C) Data are shown as mean ± SEM. Unpaired *t* test; ∗*p* < 0.05, ∗∗*p* < 0.01, ∗∗∗*p* < 0.001.

These results suggest that neither a defect in immune cell migration nor an overrepresentation of regulatory T cells in the tumor microenvironment is the cause of faster tumor growth in NLK^fl/fl^ mice.

T lymphocytes that migrate into the tumor environment and recognize tumor cells via their tumor-associated antigens can either kill their targets directly or secrete cytokines that act on tumor cells. Two important T cell effector cytokines with anti-tumor properties are TNF-α and the type II interferon IFN-γ.^17^^,^^18^^,^^19^^,^^20^^,^^21^ By analyzing the TILs, we found that NLK-deficient CD8^+^ T cells in the MC38 tumor had a decreased production of IFN-γ and TNF-α upon restimulation (Figure 4C lower image). In NLK^fl/fl^ mice, only 9.2% ± 1.1% of all tumor-infiltrating CD8^+^ cells were IFN-γ^+^, compared to 22.5% ± 3.0% (*p* < 0.001) in control animals (TNF-α^+^: NLK^fl/fl^ 4.4% ± 0.6% vs. NLK^+/+^ 10.0% ± 1.7%, *p* < 0.01). Also on a per cell level, NLK-deficient CD8^+^ TILs expressed less IFN-γ and TNF-α than their wild-type counterparts, as measured by mean fluorescence intensity (MFI) of all CD8^+^ (IFN-γ: NLK^fl/fl^ 504 ± 53 vs. NLK^+/+^ 1152 ± 151, *p* < 0.001, TNF-α: NLK^fl/fl^ 275 ± 22 vs. NLK^+/+^ 401 ± 39, *p* < 0.01). The expression of these cytokines, as well as IL-2, was also reduced in CD4^+^ TILs of NLK^fl/fl^ mice (Figure 4C upper image, Table S2).

To gain more insight into the expression profile of CD8^+^ TILs, we performed bulk RNA sequencing (RNA-seq). The mice in this experiment exhibited the described phenotype, with NLK^fl/fl^ animals developing larger tumors on average than wild-type mice (Figure 4D). Principal component analysis (PCA) showed that NLK-deficient CD8^+^ TILs were transcriptionally distinct from wild-type cells (Figure 4E). Interestingly, the wild-type mice whose TILs most closely resembled those of the knockout mice also had the largest tumors within the wild-type group (Figure 4D). A heatmap of differentially expressed genes (DEGs) showed transcriptional repression on a large scale in NLK^fl/fl^ TILs (Figure 4F). Notably, T cells deficient in NLK were found to downregulate several genes associated with cytotoxicity including *Ifng*, *Tnf*, *Gzmb*, *Prf1*, and *Fasl* (Figure 4G). We also performed gene set enrichment analysis (GSEA) to identify altered pathways using the Kyoto Encyclopedia of Genes and Genomes (KEGG) pathway database. Indeed, genes in pathways related to T cell receptor signaling and cytokine-cytokine receptor interaction were underrepresented in NLK^fl/fl^ CD8^+^ TILs (Figure 4H).

Altogether, these data suggest that NLK-deficient T cells have reduced effector functions in the tumor microenvironment.

### NLK-deficient tumor-infiltrating CD8^+^ T cells upregulate inhibitory markers

These results prompted us to investigate whether Nlk^fl/fl^ tumor-infiltrating CD8^+^ T cells show enhanced signs of T cell dysfunction. Indeed, CD8^+^ TILs from MC38 tumors of T cell-specific NLK-deficient mice expressed higher amounts of markers associated with T cell dysfunction (PD-1, TIM-3, LAG-3, and CD39) on a per cell level, depicted as MFI. Furthermore, the fraction of CD8^+^ cells expressing these markers was higher in the tumors of NLK-deficient mice (Figure 5A and Table S3). Notably, despite their cytokine defect, enhanced expression of inhibitory markers was not detectable on CD4^+^ TILs (not shown).

**Figure 5:**
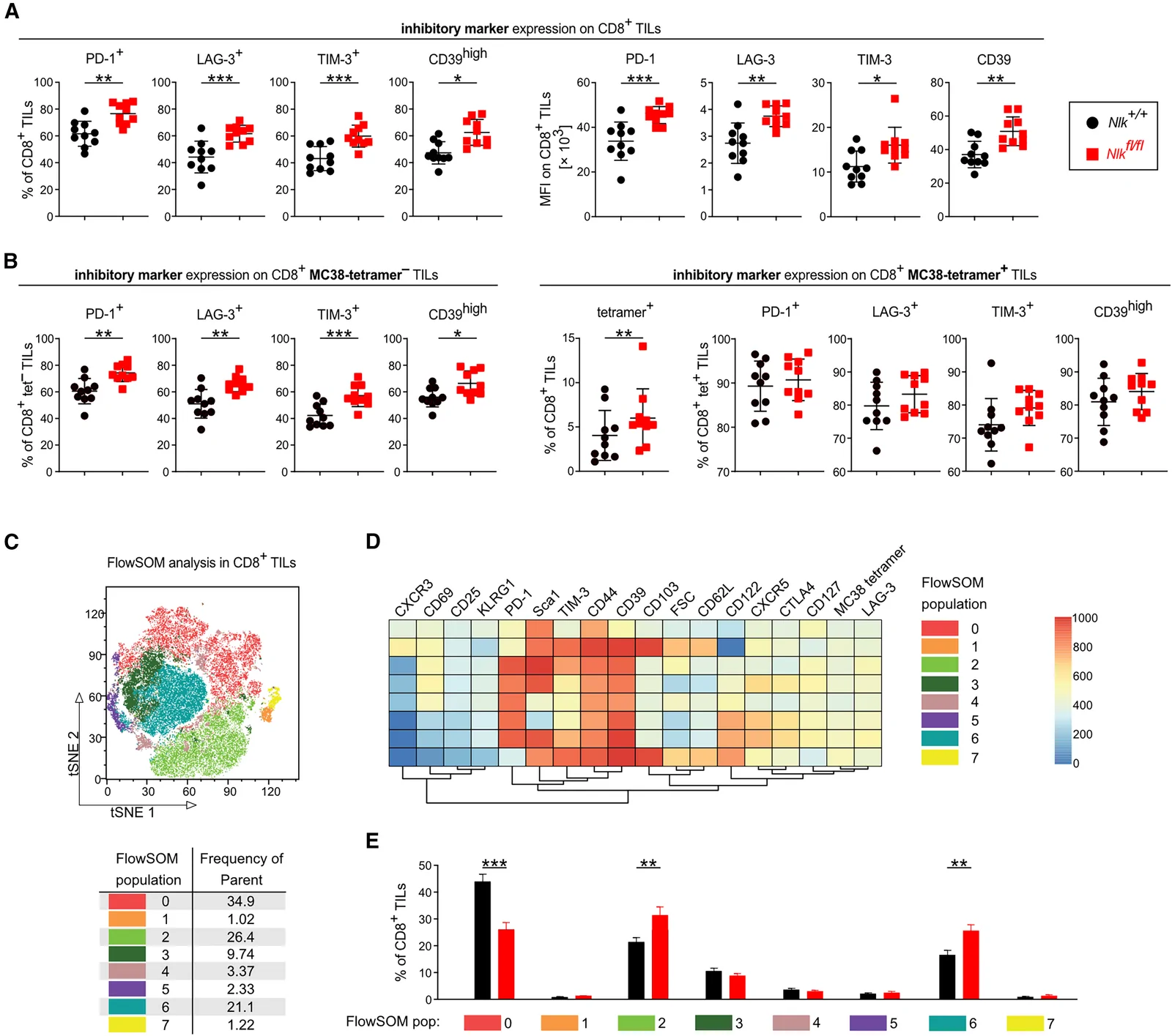
Inhibitory markers are upregulated on NLK-deficient CD8^+^ tumor-infiltrating lymphocytes Flow cytometric analyses were performed three weeks after MC38 injection, *n* = 10. (A) Staining of inhibitory markers on CD8^+^ T cells. (B) Staining of inhibitory markers gated on MC38-tetramer-negative (left) and -positive (right) CD8^+^ T cells. (C–E) Equal numbers of gated CD8^+^ TILs of ten NLK^+/+^ and ten NLK^fl/fl^ mice were concatenated in one file and unsupervised clustering was performed using the FlowSOM algorithm in FlowJo (facilitating the parameters depicted in [D] and Figure S8). (C) FlowSOM populations depicted in a t-SNE plot along with their frequencies. (D) Heatmap showing mean fluorescence intensity (MFI) of the used markers. (E) Distribution of populations in NLK^+/+^ and NLK^fl/fl^ mice. (A and B) Data are shown as mean ± SEM. *t* test; ∗*p* < 0.05, ∗∗*p* < 0.01, ∗∗∗*p* < 0.001.

We also stained CD8^+^ TILs with an MHC-I-tetramer bound to the Rpl18 neoepitope KILTFDRL, which has been shown to dominate the CD8^+^ T cell response to MC38 cells.^22^ Interestingly, the enhanced expression of inhibitory markers was only significant in MC38-tetramer-negative TILs (Figure 5B, left image). In tetramer-positive TILs, a consistent but non-significant trend was observed (Figure 5B, right image).

Notably, there were also more CD69 expressing NLK-deficient CD8^+^ T cells in the tumors (Figure 4B). CD69 is typically regarded as an early activation marker of T cells; however, it has been shown that CD69 expression on T cells can weaken the anti-tumor immune response.^23^

To better understand the nature of the infiltrating CD8^+^ T cells and the expression of different markers, we performed unsupervised clustering, which yielded distinct populations (Figures 5C–5E, S6, S7, and S8). The distribution of these populations was altered between wild-type and NLK-deficient cells: in line with MFIs and percentages based on all CD8^+^ TILs, we observed that especially populations 2 and 6 were significantly increased in the knockout group (Figure 5E). These populations are characterized by high MFIs of PD1, TIM-3, and CD39. Conversely, the population of TILs expressing low levels of PD-1, TIM-3, and CD39 (population 0) was reduced in NLK^fl/fl^ mice. These data suggest that, in a murine tumor model, NLK deficiency leads to a more pronounced expression of markers associated with CD8^+^ T cell dysfunction. Notably, blocking the PD-1/PD-L1 interaction by injecting an anti-PD-L1 antibody partially restored the anti-tumor functions of NLK-deficient T cells (Figure S9A). This may reflect the fact that the TIL population defined by PD-1^+^TIM-3^−^ is not different between wild-type and NLK^fl/fl^ mice (Figure 9B). This subset is thought to respond to anti-PD-1 therapy, in contrast to PD-1^+^TIM-3^+^ T cells,^24^^,^^25^ which are more abundant in TILs of NLK-deficient mice (Figure S9B). It should be noted that injection of the IgG2b control antibody alone resulted in delayed tumor growth. Therefore, in this experimental setting, we challenged the mice with twice the amount of tumor cells compared with our standard experiments. This distinct experimental setup altered the overall tumor growth kinetics and attenuated the genotype-dependent difference, although the direction of the effect remained consistent with that observed in our primary experiments (*p* = 0.08).

In order to evaluate NLK expression during prolonged activation, we repeatedly stimulated CD8^+^ T cells for five days and screened for differentially regulated genes using RNA-seq. Interestingly, we found that *Nlk* is downregulated^26^ (Figure S10), whereas the expression of TOX, PD-1, TIGIT, and TIM-3 increased. Additionally, TCF-1 expression decreased during repeated stimulation of CD8^+^ T cells.^27^^,^^28^^,^^29^^,^^30^^,^^31^

### NLK function is conserved in primary human T cells

Based on the results obtained with NLK-deficient murine T cells, we aimed to assess the function of NLK more directly and, at the same time, extend the results to the human setting. To this end, we enforced expression of NLK in primary human T cells by lentiviral transduction of constructs containing the gene for *NLK* (HA-tagged) or β-galactosidase (amino acids [aa] 2–83) as control. For selection purposes, a puromycin resistance gene was included (Figure 6A).

**Figure 6:**
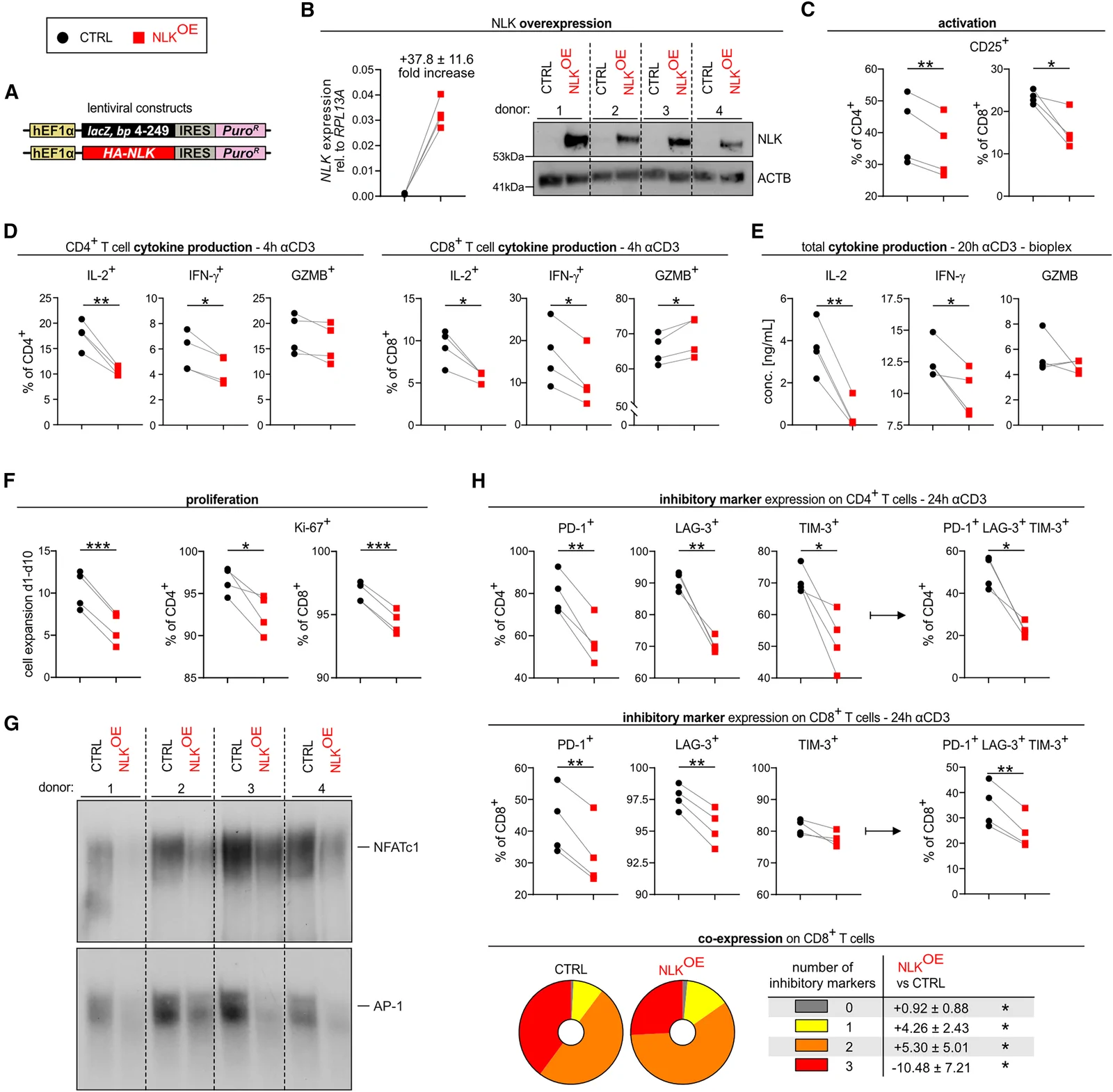
NLK restrains acute human T cell activation and effector functions Primary human CD3^+^ T cells were lentivirally engineered to express either NLK (red) or a control construct (black), followed by puromycin selection for NLK transgene expressing cells. Modified T cells were subsequently subjected to phenotypic and functional analyses. (A) Schematic overview of constructs used for NLK overexpression or corresponding control. (B) Confirmation of NLK overexpression by RT-qPCR on day 11, after 20 h restimulation via 5 μg/mL plate-bound anti-CD3 antibodies and western blot without restimulation. β-actin (ACTB) was used as a loading control. (C) Flow cytometric analysis of CD25 expression on day 10, at the end of production. (D) Intracellular flow cytometric analysis of cytokine production (IL-2, IFN-γ, and GZMB) on day 10 upon 4 h restimulation via 5 μg/mL anti-CD3 antibodies in the presence of brefeldin A and monensin added 45 min post re-stimulation. (E) Bioplex analysis on day 11, after 20 h restimulation via 5 μg/mL plate-bound anti-CD3 antibodies measuring cytokine concentrations from supernatants. (F) Proliferation analysis: Fold change of cell counts during expansion phase after transduction with NLK transgene between day 1 and 10 (left) together with flow cytometric analysis of Ki-67 expression on day 1, after 20 h restimulation via 5 μg/mL plate-bound anti-CD3 antibodies (right). (G) Equal amounts of nuclear extracts of human CD3^+^ T cells were analyzed by electrophoretic mobility shift assays to assess DNA binding of NFATc1 and AP-1 on day 11, after 20 h restimulation via 5 μg/mL plate-bound anti-CD3 antibodies. (H) Flow cytometric analysis of inhibitory marker (co-)expression on day 11, after 24 h restimulation via 5 μg/mL plate-bound anti-CD3 antibodies. All data originate from *n* = 4 donors and are represented as connected individual donor data points. Paired *t* test; ∗*p* < 0.05, ∗∗*p* < 0.01, ∗∗∗*p* < 0.001.

Overexpression of NLK was confirmed by RT-qPCR and western blot (Figure 6B). After stimulation, NLK-overexpressing human T cells were less activated, showed diminished proliferation and produced less IL-2 and IFN-γ (but not granzyme B) (Figures 6C–6F). This was accompanied by a marked reduction of NFATc1 and AP-1 binding to their respective DNA-binding sites in all four donors, as demonstrated by electrophoretic mobility shift assays (Figure 6G).

Furthermore, these cells expressed lower levels of the inhibitory receptors PD-1, LAG-3, and TIM-3 and showed significantly less co-expression of these markers (Figure 6H), a sign of less dysfunctional T cells.^32^ These data confirm our murine knockout results and demonstrate that NLK is also a negative regulator of T cell activation in the human compartment. To evaluate the function of NLK in tumor immunity, we assessed the tumor-killing capacity of NLK-overexpressing chimeric antigen receptor (CAR) T cells. To this end, we lentivirally transduced human T cells with a CAR construct consisting of the scFv derived from cetuximab (targeting the EGF receptor, EGFR) linked to CD28 and CD3ζ signaling domains (Figure 7A). Successful generation of CAR T cells was confirmed by flow cytometry. On a per cell level, CAR expression was not significantly different between the groups (not shown). NLK-overexpressing and control CAR T cells were co-cultured with EGFR^+^ A549 human lung cancer cells, engineered to stably express green fluorescent protein (GFP) and lack β2-microglobuline to prevent confounding alloreactive killing. CAR-positive cells were sorted and equal numbers were used in the experiments. GFP expression allowed us to trace tumor cell elimination by live-cell imaging. As shown in Figure 7B, NLK overexpression significantly enhanced the ability of CAR T cells to kill their target cells. Although NLK overexpression led to diminished T cell numbers by the end of the co-culture, which is consistent with the observed proliferation defect, we observed strongly increased concentrations of granzyme B in the supernatants, suggesting increased cytotoxicity of these cells (Figures 7C and 7D). Indeed, under sustained antigenic stress with A549 targets replenished every 2 days for 10 days, NLK OE-CAR T cells maintained activation and largely overcame their initial cytokine defect (Figures 7E–7G).

**Figure 7:**
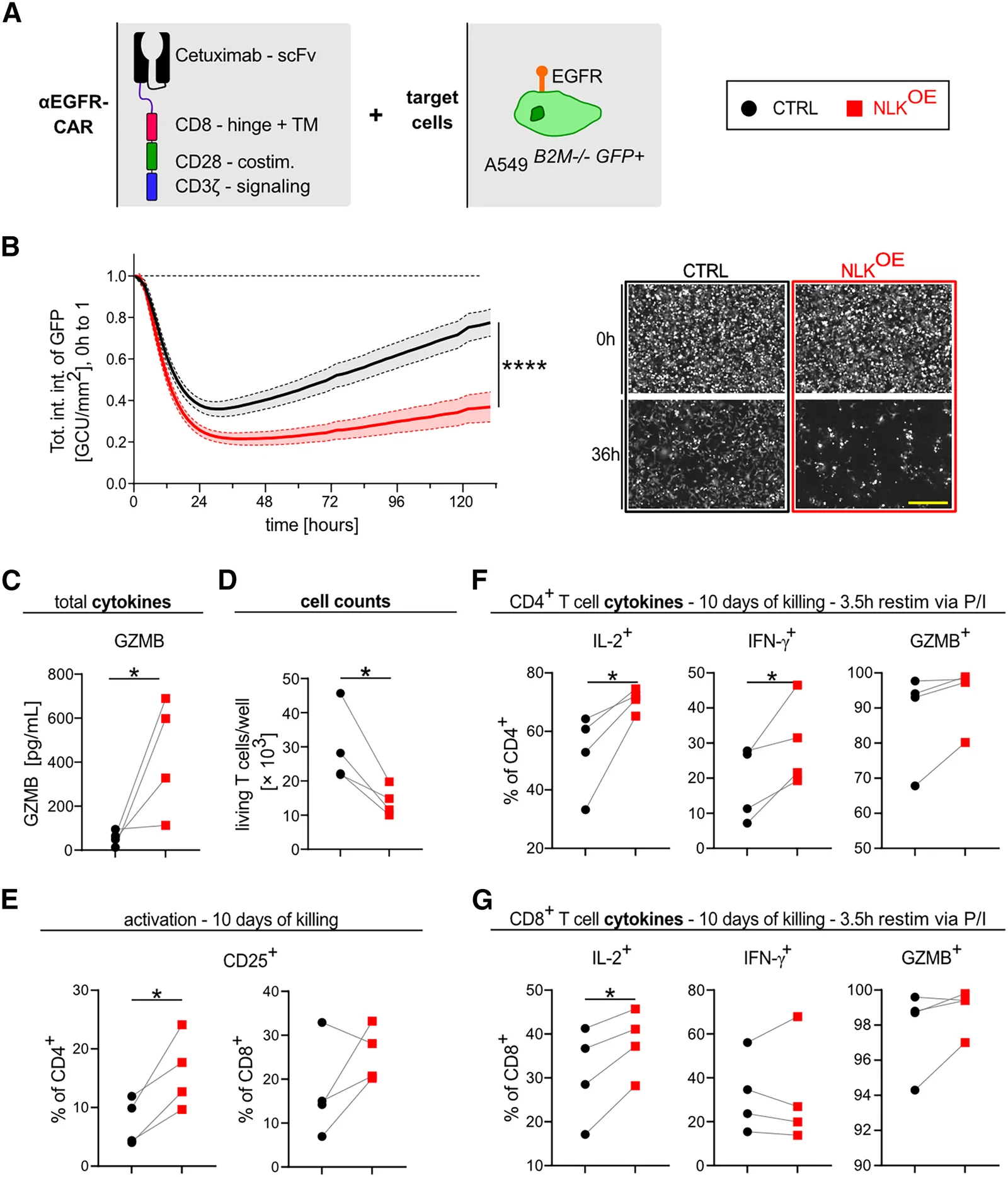
NLK preserves cytotoxic capacity during chronic stimulation of CAR-T cells Primary human CD3^+^ T cells were lentivirally engineered to express an anti-EGFR CAR together with either NLK (red) or a control construct (black), followed by puromycin selection for NLK transgene and MACS-based enrichment of CAR-expressing cells. Modified T cells were subsequently subjected to phenotypic and functional analyses. (A) Scheme of CAR and target cell model system. (B) Killing assay: NLK-modified CAR T cells were co-cultured with A549^eGFP^*B2M*^−/−^ target cells at an effector-to-target (E:T) ratio of 4:1. Tumor cell killing was monitored by live-cell imaging every 2 h. Left: killing curves. Tot. int. int. = total integrated intensity; GCU = green channel units. Right: representative microscopic fluorescent figure at start (0) and after 36 h of co-culture (scale bars, 400 μM). (C) Bioplex analysis measuring GZMB concentrations from supernatants at the end of co-culture (6 days of killing). (D) Cell counts of viable T cells per well at the end of co-culture (6 days of killing) determined by flow cytometry. (E–G) Analysis of activation and cytokine production abilities after stress test, where *NLK*-modified CAR T cells were co-cultured with A549^eGFP^*B2M*^−/−^ target cells at an effector-to-target (E:T) ratio of 4:1 with target cells being replenished every 48 h for in total 10 days. (E) Flow cytometric analysis of CD25 expression. (F and G) Intracellular flow cytometric analysis of cytokine production (IL-2, IFN-γ, and GZMB) of CD4^+^ and CD8^+^ cells on day 10 upon 3.5 h restimulation via P/I in the presence of brefeldin A and monensin added 30 min post re-stimulation. All data originate from *n* = 4 donors and are represented as connected individual donor data points. Paired *t* test (or mixed-effect analysis followed by multiple comparisons at every time point controlled by false discovery rate [FDR] for killing shown in [B]); ∗*p* < 0.05, ∗∗∗∗*p* < 0.0001.

These results demonstrate that NLK expression preserves human T cell function during long-term activation, thereby enhancing their capacity to kill tumor cells.

## Discussion

Both non-canonical Wnt/Ca^2+^ signaling and T cell receptor signaling involve the same proximal biochemical events: activation of phospholipase C (PLC) and subsequent breakdown of phosphatidylinositol 4,5-bisphosphate (PIP_2_) into diacylglycerol (DAG) and inositol trisphosphate (IP_3_). IP_3_ eventually opens calcium channels in the endoplasmic reticulum, resulting in a rapid increase in the cytosolic Ca^2+^ concentration. NLK has been shown to be activated by Ca^2+^ via calmodulin-dependent protein kinase II (CaMKII) in the non-canonical Wnt pathway, thereby antagonizing Wnt/β-catenin/TCF signaling.^33^^,^^34^ Given the importance of calcium flux in T cell activation, we speculated that NLK, which is prominently expressed in T cells, might also play a role in T cell receptor signaling. Few studies have addressed NLK functions in T cells. Fleskens et al. reported that NLK sustains Foxp3 transcriptional activity and thereby maintains the suppressive function of regulatory T cells.^14^ In the study of Daams et al., the deletion of NLK resulted in a decrease of CD8^+^ single-positive T cells in the thymus due to increased cell death in this population.^15^ However, to our knowledge, functions of NLK in mature, peripheral effector T cells have not been elucidated so far.

To address this question, we generated a mouse line with a conditional *Nlk* deletion in the T cell lineage by crossing CD4-Cre mice with floxed *Nlk* mice. T cell-specific *Nlk* knockout mice developed normally and had a normal lifespan. Their weight over time was not significantly different from that of control mice, nor was the size of their lymphoid organs. Thymocyte development appeared normal. Notably, and in contrast to Daams et al.,^15^ we did not find a reduced single-positive CD8 compartment in the thymus of NLK^fl/fl^ mice. However, it should be noted that Daams et al. used the Cre recombinase gene under the control of the Lck promoter to delete *Nlk* in the T cell lineage. Lck-Cre is already expressed in early T cell precursors, whereas CD4-Cre is not expressed before the double-positive state of thymocytes, which may explain the differences between the two studies.^35^

In peripheral lymphoid organs, the loss of NLK did not alter the numbers or distribution of T cell subsets in 12-week-old mice, with one exception: we observed significantly fewer CD62L^low^ CD44^high^ effector memory CD8^+^ T cells on a percentage basis in the periphery of healthy NLK^fl/fl^ mice compared to their wild-type counterparts up to 28 weeks of age. However, the absolute number of effector memory CD8^+^ T cells was not significantly altered due to a slight increase of the total CD8^+^ subset in NLK-deficient mice.

In contrast to a report by Fleskens et al.,^14^ we did not observe a decreased CD4^+^CD25^+^Foxp3^+^ T_reg_ subset in middle-aged NLK^fl/fl^ mice, and *in vitro* differentiation of T_regs_ was also normal. It should be emphasized that Fleskens et al. used Foxp3-Cre to generate *Nlk*-deficient T_regs_. Whether this different strategy to conditionally delete *Nlk* (as compared to our CD4-Cre approach) contributes to these conflicting results remains unclear. Nevertheless, we found that NLK deficiency reduced the ability of T_regs_ to suppress wild-type effector T cells, which is in line with Fleskens et al.^14^

By analyzing the impact of *Nlk* deletion on T cell activation, we found that mature CD4^+^ and CD8^+^ T cells lacking NLK were initially hyperactivated early after stimulation via the T cell receptor complex and CD28. They proliferated more vigorously and produced higher levels of IFN-γ and IL-2. Consistent with this phenotype, NLK-deficient CD8^+^ T cells showed increased NFAT nuclear translocation and enhanced binding of NFAT and AP-1 (c-Fos/c-Jun) to their respective consensus DNA motifs. Upon T cell stimulation, NFAT is dephosphorylated and translocates to the nucleus, where it cooperates with AP-1 to produce IL-2 and IFN-γ^36^^,^^37^^,^^38^ as well as activate T cell proliferation.^39^^,^^40^

Based on these *in vitro* findings, we initially hypothesized that NLK might function as an intracellular immune checkpoint whose inhibition could enhance antitumor immunity. Unexpectedly, however, tumor growth was less well controlled in T cell-specific *Nlk* knockout mice than in wild-type animals. This counterintuitive result was observed in two transplanted tumor models and an autochthonous MCA-induced cancer model. Because NLK deficiency in our strain is largely restricted to the T cell compartment, the accelerated tumor growth in NLK^fl/fl^ mice is therefore best explained by a defect in T cell-mediated tumor control. It is noteworthy, however, that we also observed a small but significant relative reduction in tumor-infiltrating NK cells in NLK^fl/fl^ mice. While off-target Cre activity in NK cells has not been formally demonstrated for the CD4Cre line, it is well recognized that transgenic Cre lines can exhibit recombination in unintended cell types due to incomplete representation of regulatory elements.^41^ However, murine NK cells do not express CD4, so direct off-target deletion in this population is unlikely. Instead, the reduction in NK cell infiltration is consistent with an indirect, T cell-mediated mechanism: T cells are well-established regulators of NK cell recruitment and activation within the tumor microenvironment, for example through cytokine secretion (e.g., IL-2 and IFN-γ).^42^^,^^43^ Thus, our observations are consistent with the interpretation that loss of NLK in T cells impairs functions that are required for sustained NK cell accumulation in the tumor. It should be noted, however, that the reduction in intratumoral NK cell numbers in NLK^fl/fl^ mice is not statistically significant in absolute numbers.

In line with the enhanced tumor growth observed in NLK^fl/fl^ mice, tumor-infiltrating CD8^+^ T cells lacking NLK showed markedly reduced expression of genes involved in T cell receptor signaling and cytotoxicity and produced lower levels of TNF-α and IFN-γ, two cytokines with well-established antitumor functions.^17^^,^^18^^,^^19^^,^^20^^,^^21^ Moreover, flow cytometric analysis revealed that NLK-deficient CD8^+^ T cells in the tumor microenvironment expressed higher levels of the inhibitory receptors PD-1, TIM-3, LAG-3, and the enzyme CD39. These surface proteins are particularly associated with T cell dysfunction.^27^^,^^31^^,^^44^^,^^45^ This enhanced expression of inhibitory markers was only significant in MC38-tetramer-negative TILs. There was still a trend in tetramer-positive TILs, albeit without statistical significance. Tetramer-positive cells represent a defined population recognizing the immunodominant Rpl18 neoepitope.^22^ The level of inhibitory markers is already high in the wild-type, which may limit dynamic range, while the relatively small size of this population limits statistical power.^46^ Conversely, tetramer^−^ TILs comprise a heterogeneous population of T cells with specificities for antigens that activate at different strengths.^47^ These results support a broad effect of NLK deficiency on the CD8^+^ TIL compartment but do not establish selective dysfunction of the dominant tetramer-defined population.

It should be noted that PD-1, TIM-3, LAG-3, and CD39 are often referred to as “exhaustion markers.” However, some of these proteins are also induced upon T cell receptor stimulation and are likewise considered “activation markers.” A distinguishing feature of T cell exhaustion is the duration of marker expression, as well as the co-expression of multiple markers.^48^ Whether NLK-deficient TILs are in a true “exhausted” state remains to be determined in future studies.

Importantly, overexpression of NLK in primary human T lymphocytes confirmed the results obtained in the murine models. Specifically, T cells with forced NLK expression were less activated, had a proliferation defect and produced less cytokines than the control cells. Intriguingly, however, NLK-overexpressing human CAR T cells were superior at killing their target tumor cells. This was even true despite a significant lower T cell number in the co-culture which probably reflected the diminished proliferation of NLK-overexpressing T cells. During the killing assay, however, these cells had overcome their initial activation defect and produced more granzyme B, which is consistent with their enhanced killing capacity. Overall, these data show that although NLK negatively regulates early murine and human T cell activation, it plays a crucial role in maintaining T cell function in the context of cancer immunity.

In summary, we demonstrate that NLK activity has an unexpected and previously unrecognized dual effect in murine and human T cells. Early after stimulation, NLK attenuates T cell activation. However, during chronic stimulation (such as in the context of antitumor immunity), it helps to preserve prolonged effector functions. In light of these results, one could consider the forced overexpression of the *NLK* gene in T cells (e.g., using the CRISPRa method) to delay the development of T cell dysfunction in the context of anti-tumor immunity. As diminished effector functions or persistence of intratumoral T cells are also obstacles in current immunotherapeutic regimens, combining CAR-T cell or checkpoint therapy with enhanced NLK expression could be a promising combinatorial approach.

### Limitations of the study

Several limitations should be considered when interpreting these findings. First, we have not defined “T cell dysfunction” in detail. In particular, we have not established whether these cells are truly immunologically exhausted. Consequently, we did not perform a subset-resolved analysis of TILs, e.g., progenitor versus terminal exhausted T cells (Tex). Future studies employing established precursor/terminal Tex gating strategies will be needed to address this gap. Second, due to a lack of subset-specific Cre-drivers, we were unable to analyze the contribution of CD4^+^ and CD8^+^ T cells to the tumor phenotype separately. However, NLK overexpression in human T cells clearly showed that NLK has important functions in both subsets. Third, enhanced expression of inhibitory markers was only significant in MC38-tetramer-negative TILs. Therefore, our study does not imply that the observed dysfunctional phenotype primarily reflects impairment of tumor-reactive T cells. Future studies should also involve the additional functional characterization of tetramer-negative and -positive TILs. Fourth, as only female mice were used in the tumor experiments, it was not possible to evaluate the influence of sex on the reported phenotypes. Fifth, the mechanistic relationship between NLK and NFAT/AP-1 was not resolved but will be the subject of future studies. Finally, forced overexpression may exceed physiological levels and may therefore not fully represent the endogenous situation. Nevertheless, the data obtained with NLK overexpressing T cells are consistent with the complementary knockout data.

## Resource availability

### Lead contact

Requests for further information and resources should be directed to and will be fulfilled by the lead contact, Gruber T (thomas.gruber@i-med.ac.at).

### Materials availability

All unique/stable reagents generated in this study are available from the lead contact without restriction.

### Data and code availability

• RNA-seq data have been deposited at GEO under accession no. GSE330033 and are publicly available as of the date of publication.
• Data reported in this paper will be shared by the lead contact upon request.
• This paper does not report original code.
• Any additional information required to reanalyze the data reported in this paper is available from the lead contact upon request.

## Acknowledgments

We would like to thank Martina Sykora for her help in setting up the 25-parameter Aurora panel (this panel will be published separately as a method paper), Nina Posch and Friedrich Fresser for technical assistance, and the 10.13039/501100009980Medical University of Innsbruck for providing facilities. This research was funded in whole or in part by the 10.13039/501100002428Austrian Science Fund (10.13039/501100002428FWF) (10.55776/PAT9292223 and 10.55776/T1292). For open access purposes, the author has applied a CC BY public copyright license to any author-accepted manuscript version arising from this submission.

## Author contributions

All authors have contributed substantially to the work. S.P. was involved in all the experiments, analysis, and manuscript preparation. J.K. helped with statistics and analysis of RNA-seq data. He also performed overexpression experiments with human T cells and prepared final figures. N.T. performed T_reg_ assays. K.S. conceptualized and helped with the immune phenotyping as well as the T_reg_ assay and *in vitro* repeated stimulation assay. V.K. helped with MCA injections and together with D.H. helped with repeated *in vitro* stimulations. W.O., M.P., and P.B. were involved in setting up TIL FACS panels, TIL experiments, and analysis. K.A.-S. helped with MCA injections and TIL experiments. T.G. prepared the manuscript, performed experiments and analysis and, together with G.B., provided the study conception and the overall supervision of the research at all stages.

## Declaration of interests

The authors declare no competing interests.

## Declaration of generative AI and AI-assisted technologies in the manuscript preparation process

The authors used DeepL Write and Academic AI during the preparation of this work to refine the phrasing and improve the clarity and readability of the manuscript.

## STAR★Methods

### Key resources table

**Table:** 

REAGENT or RESOURCE	SOURCE	IDENTIFIER
**Antibodies**		

InVivoMab anti-mouse PD-L1 (B7-H1)	BioXcell	Cat# BE0101; RRID: AB_10949073
InVivoMAb rat IgG2b isotype control, anti-keyhole limpet hemocyanin	BioXcell	Cat#BE0090; RRID: AB_1107780
InVivoMAb anti-mouse CD3ε (Clone: 145-2C11)	BioXcell	Cat#BE0001-1; RRID: AB_1107634
InVivoMab anti-mouse CD28	BioXcell	Cat#BE0015-1; RRID: AB_1107624
InVivoMAb anti-human CD3 (clone OKT3)	BioXcell	Cat#BE0001-2; RRID: AB_1107632
NLK (D9X3C) Rabbit mAb	CellSignaling	Cat#94350; RRID: AB_2800227
Goat anti-mouse-HRP	N/A	Cat#31430; RRID: AB_228307
Goat anti-rabbit-HRP	N/A	Cat#31460; RRID: AB_228341
β-Actin (C-4)	SantaCruz	Cat#sc-47778; RRID: AB_626632
NFATc1 (7A6)	SantaCruz	Cat# sc-7294; RRID: AB_2152503
Anti-Lamin B1	Abcam	Cat#ab16048; RRID: AB_443298
TruStain FcX(TM) (anti-mouse CD16/32)	Biolegend	Cat#101320; RRID: AB_1574975
BD OptiBuild™ BB700 Hamster Anti-Mouse CD103 (2E7)	BD	Cat# 748240; RRID: AB_2872671
CD11b (cloneM1/70) BB660	BD	Custom conjugation
BD OptiBuild™ BV750 Rat Anti-Mouse CD122 (5H4)	BD	Cat# 747444; RRID: AB_2872124
BD Pharmingen™ PE-Cy™7 Rat Anti-Mouse CD127 (SB/199)	BD	Cat#560733; RRID: AB_1727424
PE/Cyanine5 anti-mouse CD19 Antibody (6D5)	Biolegend	Cat# 1155-09L; RRID: AB_2794651
PE/Cyanine7 anti-mouse CD19 Antibody (6D5)	Biolegend	Cat#115520; RRID AB_313655
BD Horizon™ BV605 Rat Anti-Mouse CD25 (PC61))	BD	Cat# 563061; RRID: AB_2737982
APC anti-mouse CD25 Antibody (PC61)	Biolegend	Cat#102011; RRID: AB_312860
PE anti-mouse CD25 Antibody (PC61)	Biolegend	Cat# 102008; RRID: AB_312857
BD Horizon™ BUV737 Hamster Anti-Mouse CD3e (145-2C11)	BD	Cat#612771; RRID: AB_2870100
Pacific Blue™ anti-mouse CD3 Antibody (17A2)	Biolegend	Cat#100214; RRID: AB_493645
PE anti-mouse CD39 Antibody (Duha59)	Biolegend	Cat#143804; RRID: AB_11218603
Brilliant Violet 570™ anti-mouse CD4 Antibody (RM4-5)	Biolegend	Cat# 100541; RRID: AB_10897943
BD Horizon™ V500 Rat anti-Mouse CD4 (RM4-5)	BD	Cat# 560782; RRID: AB_1937315
FITC anti-mouse CD4 Antibody (RM4-5)	Biolegend	Cat# 100510; RRID: AB_312713
BD Horizon™ V500 Rat anti-Mouse CD4 (RM4-5)	BD	Cat# 560782; RRID: AB_1937315
BD Pharmingen™ PE Rat Anti-Mouse CD4 (RM4-5)	BD	Cat# 553049; RRID: AB_394585
BD Horizon™ BV786 Rat Anti-Mouse CD44 (IM7)	BD	Cat# 563736; RRID: AB_2738395
APC/Cyanine7 anti-mouse/human CD44 Antibody (IM7)	Biolegend	Cat# 103028; RRID: AB_830785
PE/Cyanine7 anti-mouse/human CD44 Antibody (IM7)	Biolegend	Cat# 103030; RRID: AB_830787
FITC anti-mouse/human CD44 Antibody	Biolegend	Cat# 103006; RRID: AB_493685
BD Pharmingen™ APC-Cy™7 Rat Anti-Mouse CD45 (30-F11)	BD	Cat# 557659; RRID: AB_396774
BD Horizon™ BUV805 Mouse Anti-Mouse CD45.2 (104)	BD	Cat# 741957; RRID: AB_2871265
BUV496 Rat Anti-Mouse CD62L (MEL-14)	BD	Custom conjugation
BD Pharmingen™ APC Rat Anti-Mouse CD62L (MEL-14)	BD	Cat# 553152; RRID: AB_398533
FITC anti-mouse CD69 Antibody (H1.2F3)	Biolegend	Cat# 104506; RRID: AB_313109
BD Horizon™ BUV395 Rat Anti-Mouse CD8a (53–6.7)	BD	Cat# 563786; RRID: AB_2732919
PerCP/Cyanine5.5 anti-mouse CD8a Antibody (53–6.7)	Biolegend	Cat# 100734; RRID: AB_2075238
BD Horizon™ V500 Rat anti-Mouse CD8a (53–6.7)	BD	Cat# 560778; RRID: AB_1937329
PerCP/Cyanine5.5 anti-mouse CD8a Antibody (53–6.7)	Biolegend	Cat# 100734; RRID: AB_2075238
BD Pharmingen™ APC Rat Anti-Mouse CD8a (53–6.7)	BD	Cat# 553035; RRID: AB_398527)
BD Horizon™ APC-R700 Hamster Anti-Mouse CD152/CTLA-4 (UC10-4F10-11)	BD	Cat# 565778; RRID: AB_2739350
Brilliant Violet 510™ anti-mouse CD183 (CXCR3) Antibody (CXCR3-173)	Biolegend	Cat# 126527; RRID: AB_2562204
APC/Cyanine7 anti-mouse CD185 (CXCR5) Antibody (L138D7)	Biolegend	Cat# 145525; RRID: AB_2566798
FOXP3 Monoclonal Antibody (FJK-16s), FITC, eBioscience™	ThermoFisher	Cat# 11–5773-82; RRID: AB_465243
FOXP3 Monoclonal Antibody (NRRF-30), PE, eBioscience™	ThermoFisher	Cat# 12–4771-82; RRID: AB_529580
Granzyme B Monoclonal Antibody (NGZB), PE, eBioscience™	ThermoFisher	Cat# 12–8898-82; RRID: AB_10870787
BD Pharmingen™ APC Rat Anti-Mouse IL-2 (JES6-5H4)	BD	Cat# 554429; RRID: AB_398555
PE/Cyanine7 anti-mouse IFN-γ Antibody (XMG1.2)	Biolegend	Cat# 505826; RRID: AB_2295770
BD Horizon™ BV711 Hamster Anti-Mouse KLRG1 (2F1)	BD	Cat# 564014; RRID: AB_2738542
BB790P Rat Anti-Mouse CD223/Lag3 (C9B7W)	BD	Custom conjugation
BD OptiBuild™ BUV661 Mouse Anti-Mouse NK-1.1 (PK136)	BD	Cat# 741477; RRID: AB_2870942
CD314 (NKG2D) Monoclonal Antibody (CX5), APC, eBioscience™	ThermoFisher	Cat# 17–5882-82; RRID: AB_469464
BD Horizon™ PE-CF594 Hamster Anti-Mouse CD279 (PD-1) (J43)	BD	Cat# 562523; RRID: AB_2737634
Pacific Blue™ anti-mouse Ly-6A/E (Sca-1) Antibody	Biolegend	Cat# 108119; RRID: AB_493274
PE/Cyanine7 anti-mouse TCR β chain Antibody (H57-597)	Biolegend	Cat# 109221; RRID: AB_893627
BD OptiBuild™ BV650 Mouse Anti-Mouse CD366 (TIM-3) (5D12/TIM-3)	BD	Cat# 747623; RRID: AB_2744189
PerCP/Cyanine5.5 anti-mouse TNF-α Antibody (MP6-XT22)	Biolegend	Cat# 506322; RRID: AB_961434
Pacific Blue™ anti-human CD4 Antibody (clone OKT4)	Biolegend	Cat#317424; RRID: AB_571953
Brilliant Violet 510™ anti-human CD8a Antibody (clone RPA-T8)	Biolegend	Cat# 301048; RRID: AB_2561942
PerCP/Cyanine5.5 anti-human CD25 Antibody (clone M-A251)	Biolegend	Cat#356112; RRID: AB_2561979
PE/Cyanine7 anti-human CD223 (LAG-3) Antibody (clone 11C3C65)	Biolegend	Cat#No. 369310; RRID: AB_2629753
PE anti-human CD279 (PD-1) Antibody (clone EH12.2H7)	Biolegend	Cat#329906; RRID: AB_940483
PerCP/Cyanine5.5 anti-human CD366 (Tim-3) Antibody (clone F38-2E2)	Biolegend	Cat#345016; RRID: AB_2561934
FITC anti-human/mouse Granzyme B Antibody (clone GB11)	Biolegend	Cat#515403; RRID: AB_2114575
PE/Cyanine7 anti-human IFN-γ Antibody (clone 4S.B3)	Biolegend	Cat# 502528; RRID: AB_2123323
PE anti-human IL-2 Antibody (clone MQ1-17H12)	Biolegend	Cat#500307; RRID: AB_315094
APC anti-human Ki-67 Antibody	Biolegend	Cat# 350514; RRID: AB_10959327

**Biological samples**		

Leukocyte reduction cones/PBMCs of healthy donors	Blood bank of Innsbruck University Hospital	N/A

**Chemicals, peptides, and recombinant proteins**		

GoTaq G2 Hot Start Polymerase	Promega	Cat#M7405
5× Green GoTaq®FlexBuffer	Promega	Cat#M891A
MgCl2	Promega	Cat#A3511
PCR Nucleotide Mix	Promega	Cat#C1141
Acridine Orange/Propidium Iodide Stain	Biocat	Cat#F23001-LG
Collagenase D	Roche	Cat#11088858001
DNAse I	Roche	Cat#11284932001
EDTA	Sigma-Aldrich	Cat#03690-100ML
RPMI 1640 medium	PAN Biotech	Cat# P04-17500
FCS	Biowest	Cat# S1810-500
Penicillin/Streptomycin 10 000 U/10 mL	PAN Biotech	Cat# P06-07100
2 mM L-glutamine	PAN Biotech	Cat# P04-80050
Phorbol-12,13-dibutyrat	Sigma-Aldrich	Cat#P1269
Ionomycin calcium salt	Sigma-Aldrich	Cat#I0634
BD GolgiPlug™ Protein Transport Inhibitor (Containing Brefeldin A)	BD	Cat#555029
BD GolgiStop™ Protein Transport Inhibitor (Containing Monensin)	BD	Cat#554724
DMEM, w: 1.0 g/L Glucose, w/o: L-Glutamine, w: Sodium pyruvate, w: 3.7 g/L NaHCO3	PAN Biotech	Cat#P04-01500
3-Methylcholanthren	Sigma-Aldrich	Cat#213942
Corn oil	Sigma-Aldrich	Cat#C8267
Oligo(dT)15 Primer	Promega	Cat#C1101
RNasin® Ribonuclease Inhibitor	Promega	Cat#N2111
Pierce™ Lane Marker Reducing Sample Buffer	ThermoFisher	Cat#39000
Invitrogen™ NuPAGE™ 4 bis 12%, Bis-Tris, 1.0–1.5 mm, Mini-Protein-Gele	ThermoFisher	Cat#10472322
Immobilon-P Membran, PVDF, 0.45 μm, 26.5 cm x 3.75 m-Rolle	Merck	Cat#IPVH00010
SuperSignal™ West Pico PLUS Chemiluminescent Substrate	ThermoFisher	Cat#34580
IL-15	Biolegend	Cat#570302
IL-7	Biolegend	Cat#581902
Human recombinant IL-2	Biolegend	Cat#589102
BD Horizon™ Fixable Viability Stain 440UV	BD	566332
BD Horizon™ Fixable Viability Stain 780	BD	565388
BD Horizon™ Brilliant Stain Buffer Plus	BD	566385
Fixation Buffer	Biolegend	Cat#420801
Intracellular Staining Permeabilization Wash Buffer (10×)	Biolegend	Cat#421002
eBioscience™ Foxp3/Transcription Factor Staining Buffer Set	ThermoFisher	Cat#00–5523-00
7-AAD Viability Staining Solution	Biolegend	Cat#420404
Fetal calf serum	Biowest	Cat#S1810-500
5(6)-CFDA, SE; CFSE (5-(and-6)-Carboxyfluorescein Diacetate, Succinimidyl Ester), mixed isomers	ThermoFisher	Cat#C1157
Mitomycin C	AppliChem	Cat#A2190
Acridine Orange/Propidium Iodide stain	Biocat	Cat#F23001-LG
EMSA stabilizing solution D	Activ Motif	Cat#37488
Ethylenediaminetetraacetic acid (EDTA)	ThermoFisher	Cat#J15694.AP
Phosphate buffered saline (PBS)	ThermoFisher	CatJ62036.K3
HBSS	PAN Biotech	Cat#P04-34500
Lymphopure	Biolegend	Cat#426202
Non-essential amino acids	Sigma	Cat#M7145
Opti-MEM™ I Reduced Serum Medium	ThermoFisher	Cat#31985062
Lipofectamine 2000	ThermoFisher	Cat#11668019
Lenti-X™ Concentrator	Takara	Cat#631232
Dynabeads™ Human T-Activator CD3/CD28 for T cell Expansion and Activation	ThermoFisher	Cat#11132D
Polybren Infektions-/Transfektionsreagenz	Sigma-Aldrich	Cat#TR-1003-G
Puromycin	Sigma-Aldrich	Cat#P8833
Biotinylated recombinant EGFR	Creative Biolabs	Cat#CARD-LX025
OVA (257–264) peptide fragment SIINFEKL	Eurogentec	Cat#AS-60193–1
TGFβ	Biolegend	Cat#B781802
anti-IFNγ	eBioscience	Cat#14–7311-85
anti-IL-4	eBioscience	Cat#14–7041-85
TrypLE Express	ThermoFisher	Cat#12604021
Brilliant Violet 421™ Streptavidin	Biolegend	Cat#405225
Biotinylated Human EGFR CAR Detection Reagent, His and Avi Tag	CreativeBiolabs	Cat# CARD-LX025
g-[^32^P]-ATP	Revvity	Cat#BLU002A100UC
Methyl-^3^H-thymidine	Revvity	Cat#NET027250UC

**Critical commercial assays**		

Omniscript RT Kit (200)	Qiagen	Cat#205113
Qiagen, 74106, RNeasy Mini Kit (250)	Qiagen	Cat#74106
CD8a+ T cell Isolation Kit, mouse	Miltenyi	Cat#130–104-075
CD4^+^ T cell Isolation Kit, mouse	Miltenyi	Cat#130–104-454
CD4^+^CD25^+^ Regulatory T cell Isolation Kit, mouse	Miltenyi	Cat#130–091-041
CD8a (Ly-2) MicroBeads, mouse	Miltenyi	Cat#130–117-044
CD4 (L3T4) MicroBeads, mouse	Miltenyi	Cat#130–117-043
Anti-Biotin MicroBeads	Miltenyi	Cat#130 090 485
CD62L (L-selectin) MicroBeads, mouse	Miltenyi	Cat#130–049-701
LS columns	Miltenyi	Cat#130–042-401
QuadroMACS separator	Miltenyi	Cat#130–091-051
MojoSortTM Human CD3Cell Isolation Kit	Biolegend	Cat#480131
Bio-Plex Pro Mouse Chemokine IL-2 Set	BioRad	Cat#12002439
Bio-Plex Pro Mouse Chemokine IFN-γ Set	BioRad	Cat#12002438
Bio-Plex Pro Human Cytokine IL-2 Set	Biorad	Cat#171-B5003M
Bio-Plex Pro Human Cytokine IFN-γ Set	Biorad	Cat#171-B5019M
Granzyme B Human ProcartaPlex™ Simplex Kit	ThermoFisher	Cat#EPX01A-12027–901
TaqMan Gene Expression primer and probe NLK	ThermoFisher	AssayID Hs00939676_m1
TaqMan Gene Expression primer and probe NLK	ThermoFisher	AssayID Hs00939677_m1
TaqMan Gene Expression primer and probe IFN-γ	ThermoFisher	AssayID Mm00801778_m1
TaqMan Gene Expression primer and probe GAPDH	Fisher Scientific	Cat#10137104
TaqMan Gene Expression primer and probe RPL13A human	Thermo Fisher	AssayID Hs04194366_g1

**Deposited data**		

BulkRNA-Seq raw data of CD8^+^ TILs (FACS) from MC38 tumors harvested three weeks after injection	This paper	deposited in the Gene Expression Omnibus (GEO)under accession number GSE330033

**Experimental models: Cell lines**		

A549 B2M KO	Abcam	Cat#ab286573; RRID: CVCL_C3CC
HEK293T	Abcam	Cat#ab255449; RRID: CVCL_0063
MC38	Gift from Professor Waldner (University of Erlangen, Erlangen, Germany)	RRID: CVCL_B288
TC1	Cytion	Cat#305388; RRID:CVCL_4699

**Experimental models: Organisms/strains**		

B6;129-Nlktm1.1Ecan/J mice; common Name NLKfl	Jackson Laboratories	strain #024537; RRID:IMSR_JAX:024537
Tg(Cd4Cre)1Cwi/BfluJ mice; common name CD4Cre	Jackson Laboratories	strain #017336; RRID:IMSR_JAX:017336
C57BL/6-Tg(TcraTcrb)1100Mjb/J; common name OT-1	Jackson laboratories	strain #003831; RRID:IMSR_JAX:003831

**Oligonucleotides**		

NLKflFWD (19494) TTCAGTCGCCAGAATCAA GA	Jackson Laboratories strain #024537	Eurofins custom-synthesized
NLKflREV (19495) CCACCAAATTCTGCCTTCC	Jackson Laboratories strain #024537	Eurofins custom-synthesized
CD4cre co: AACTTGCACAGCTCAGAATGC	Eurofins	custom-synthesized
CD4cre mu: TTAGGGTGGGGCTCAGAAGG	Eurofins	custom-synthesized
CD4cre wt: ACCTGAGATTCCACCAAACTTGA	Eurofins	custom-synthesized
EMSA NFAT consensus binding site oligonucleotide: GCCCAAAGAGG AAAATTTGTTTCATACAG	Eurofins	custom-synthesized
EMSA AP-1 consensus binding site oligonucleotide:CGCTTGATGA CTCAGCCGGAA	Eurofins	custom-synthesized

**Recombinant DNA**		

LentiArt™ Virus Packaging Plasmids	Creative Biolabs	Cat# CART-027CL
pLV[CAR]-EF1A>CD8-leader/{Cetuximab}/CD8-hinge/CD8-TM/CD28-CD3zeta	Generated in this study, VectorBuilder	Vector ID: VB241014-1216jtq
pLV[Exp]-EF1A > ORF Stuffer:IRES:Puro lentiviral plasmid	Generated in this study, VectorBuilder	Vector ID: VB241126-1331hgg
pLV[Exp]-EF1A > hNLK[NM_016231.5]/HA:IRES:Puro lentiviral plasmid	Generated in this study, VectorBuilder	Vector ID: VB260129-1174nxw
pLVX-EGFP-IRES-Puro lentiviral plasmid	gift from R. Sobol, described by Fang et al.^49^	Addgene #128652

**Software and algorithms**		

FlowJo (v10.8.1. and 10.10.1)	BD	N/A
GraphPad Prism (v10.2.3)	GraphPad Software, LLC	N/A
Incucyte2022B analysis software	Sartorius	N/A
R (v4.3.3.)	R Foundation for Statistical Computing	N/A
Academic AI	Medical University of Innsbruck	N/A
DeepL Write	DeepL SE	N/A

**Other**		

FACSCalibur	BD	N/A
FACSCanto II (4-2-2 configuration)	BD	N/A
5-laser Cytek®Aurora	Cytek	N/A
Bio-Plex 200 device	Biorad	N/A
1450 Microbeta Trilux Liquid Scintillation and Luminescence Counter (Wallac)	PerkinElmer	N/A
Incucyte SX5 system	Sartorius	N/A

### Experimental model and study participant details

Primary human PBMCs were obtained from healthy donors via the Blood Bank of Innsbruck University Hospital (no identifiable patient data were provided and thus, per institutional policy, no ethics approval is required). Full anonymization precluded consideration of sex or age, which limits assessment of sex-related effects, though donors represent an unsystematic, randomized sample.

The cell lines used in this study were not authenticated and not tested for mycoplasma.

Mice were housed under specific pathogen free (SPF) conditions at the animal facility of the Medical University of Innsbruck. All animal experiments were conducted in accordance with the Austrian Animal Welfare Law and the Animal Experiments Act and were approved by the Committee of the Animal Care of the Austrian Federal Ministry of Science and Research (GZ: 2023–0.433.292). These conditions included an ambient temperature of 21–22°C, a humidity level of 50%, a 12 h light/dark cycle and *ad libitum* access to food and water. Max. 5 mice were housed per GM500 IVC cage (tecniplast green line; 501cm2). Mice were monitored daily by the animal facility personnel and additionally three times a week during the ongoing experiments for clinical signs of significant distress (using score sheets e.g., taking the body condition score in account) by the scientists. Female mice were used for the tumor experiments. Experimental mice were selected at random from litters. At study endpoints or when reaching a defined humane endpoint (death was not an endpoint), mice were euthanized by gradual-fill carbon dioxide (CO_2_) inhalation in a euthanasia chamber, using a displacement rate of 30%–70% of the chamber volume per minute; CO_2_ flow was maintained for at least 1 min after respiratory arrest (in accordance with the AVMA 2020 guidelines). Death was confirmed by cervical dislocation. Tissues (tumor, spleen, lymph nodes) were collected immediately postmortem at defined time points. No anesthesia was used for intraperitoneal, or subcutaneous injections or blood sampling. The animals were restrained by hand for intraperitoneal or subcutaneous injections. Considerations of the 3Rs: the animals were handled by the same researcher whenever possible to minimize stress and help the mice become accustomed to that researcher. Furthermore, to achieve our goal of collecting as much relevant data as possible from a single animal and thereby minimizing the number of animals used, we worked closely together as a research group, particularly during the endpoint analyses.

For T cell compartment-specific deletion, B6;129-Nlktm1.1Ecan/J mice (Jackson Laboratories strain #024537; common Name NLK^fl^) were crossed with Tg(Cd4Cre)1Cwi/BfluJ mice (Jackson Laboratories strain #017336; common name CD4Cre), containing the CD4 enhancer, promoter and silencer sequences, driving the expression of a Cre recombinase gene. The corresponding offspring with the *loxP* sites as well as CD4-driven Cre recombinase were further bred to generate control mice (NLK^+/+^/CD4Cre^+^) as well as T cell conditional knockout mice (NLK^fl/fl^/CD4Cre^+^). Unless stated otherwise, mice aged between 8 and 12 weeks were used in the experiments conducted in this study.

### Method details

#### Transplanted tumor experiments

MC38 colon carcinoma and TC-1 transformed lung epithelial cells were cultured in DMEM (PAN Biotech; P04-01500) and RPMI 1640 (PAN Biotech; P04-17500), respectively. The media were supplemented with 10% heat inactivated FCS, 100 U/mL penicillin, 100 μg/mL streptomycin and 2 mM L-glutamine at 37°C and 5% CO_2_. The cells were always split the day before injection, to reach a confluency of about 80% on the harvest day. For harvesting and passaging, adherent cells were washed once with 10 mL of warm 1× phosphate buffered saline (PBS). 3 mL of TrypLE Express (Thermo Fisher Scientific, 12604021) were used to detach cells at 37°C for two to 5 min. The enzymatic reaction was stopped, using at least 6 mL of cell culture medium. The cell suspension was transferred into a 50 mL tube (Corning, BDL352070) and centrifuged for 5 min at 300 × g at room temperature (RT). Cells were resuspended in medium for further cultivation or in PBS before being injected subcutaneously (s.c.) into the left, ventral flank of recipient mice, shaved two days prior to injection (MC38: 2.5 × 10^5^ cells in 100 μL; TC-1: 1.5 × 10^5^ cells in 100 μL). For checkpoint blockade experiments, 500.000 MC38 colon carcinoma cells were injected s.c. and 250 μg anti-PD-L1 (BioXcell # BE0101, Clone 10F.9G2) or IgG2b control (BioXcell # BE0090, Clone LTF-2) was administered i.p. twice a week. Tumor growth (length [L] and width [W]) was measured every second or third day using a caliper for up to three weeks. Tumor volume (V) was estimated assuming an ellipsoidal tumor growth: V = (L x W^2^)∗(π/6).

#### MCA tumor induction

Mice were shaved two days prior to induction at the injection site on the right hind leg. MCA (3-methylcholanthrene, Sigma-Aldrich, Cat# 213942) was dissolved at 2 mg/mL in corn oil (Sigma C8267-500 ML) and taken up in a 1 mL syringe with a 27G needle. 100 μL of this solution were injected subcutaneously and tumor growth was controlled once a week. Upon detecting palpable tumors (usually after 10–12 weeks), control intervals were shortened to two times per week. Mice were sacrificed once the tumor length exceeded 15 mm.

#### Genotyping

NLK-flox genotype was confirmed using specific primers (NLK^fl^_FWD_: TTCAGTCGCCAGAATCAAGA and NLK^fl^_REV_: CCACCAAATTCTGCCTTCC), yielding a 167bp wt amplicon and a 262bp mutant amplicon.

CD4-Cre genotype was confirmed using specific PCR primers for the Cre-tg (CD4cre co: AACTTGCACAGCTCAGAATGC; CD4cre mu: TTAGGGTGGGGCTCAGAAGG; CD4cre wt: ACCTGAGATTCCACCAAACTTGA resulting in 560bp wt fragments and 741bp mutant fragments, being able to distinguish homo- and heterozygous animals^50^).

PCR was performed with GoTaq G2 Hot Start Polymerase and the corresponding buffer, 2 mM MgCl_2_ and 200 nM dNTPs (all Promega; Cat. No. M7405, M891A, A3511, C1141) using the following cycling conditions:

**Table:** 

NLK flox PCR				CD4 Cre PCR		
Cycling Step#	Temp. °C	Time	Note	Cycling Step#	Temp. °C	Time
1	94	–	–	1	95	5 min
2	94	20 s	–	2	95	30 s
3	65	15 s	−0.5°C per cycle	3	61	30 s
4	68	10 s	–	4	72	45 s
5	repeat steps 2–4 10×			5	repeat steps 2–4 40×	
6	94	15 s	–	6	72	10min
7	60	15 s	–	7	10	hold
8	72	10 s	–			
9	repeat steps 6–8 28×					
10	72	2 min	–			
11	10	hold	–			

#### Generation of single-cell suspension, isolation of T cells and culture conditions

Single-cell suspensions from thymus, spleen or lymph nodes (superficial cervical, axillary, brachial, inguinal) were obtained by mechanical disintegration of the organs from sex and age matched animals using 100 μM cell strainers. In case of splenocytes, red blood cells were lysed for 6 min at room temperature (RT) using 0.15 M NH_4_Cl, 10 mM KHCO_3_ and 0.1 mM EDTA in a.d. followed by a wash step with PBS. Cell counts and viability were determined using LUNA Automated Cell Counter (Logos Biosystems) after staining with acridine orange/propidium iodide (Biocat; F23001-LG). CD8^+^ or CD4^+^ cells were isolated by negative selection using magnetic cell separation (MACS) (Miltenyi Biotec; 130–104-075 and 130–104-454) with LS columns and a QuadroMACS separator (all Miltenyi Biotec) following the manufacturers’ instructions. T cell purity was checked by flow cytometry.

Tumors were transferred to 2 mL Eppendorf tubes containing 1 mL tumor digestion mix (1× PBS with 2.5 mg/mL Collagenase D [Roche 11088858001], 1 mg/mL DNAse I [Roche 11284932001], 2.5 mg/mL MgCl_2_ and 2% FCS) and cut into small pieces with scissors. The tubes were filled up with tumor digestion mix. During a 45 min incubation time at 37°C with 5% CO_2_ the tubes were shaken every now and then. To prevent dendritic cell-T cell aggregates, EDTA (Sigma-Aldrich, Cat. No. 03690-100 ML) was added (0.01 M final EDTA concentration) and incubated for 5 min. With the plastic end of a 5 mL syringe plunger the mix was pushed through a 100 μm cell strainer. Afterward the cells were filtered through a 40 μm cell strainer and washed with ice-cold PBS. The final tumor cell suspension was resuspended in PBS/2% FCS.

All centrifugation steps were performed at 300 × g for 7 min at 4°C.

Cells (sorted T cells, thymocytes or splenocytes) were cultured in RPMI 1640 medium (PAN Biotech; P04-17500), supplemented with 10% heat inactivated FCS (Biowest; S1810-500), 100 U/mL penicillin, 100 μg/mL streptomycin (PAN Biotech; P06-07100) and 2 mM L-glutamine (PAN Biotech; P04-80050) at 37°C and 5% CO_2_.

#### *In vitro* polyclonal stimulation

For polyclonal stimulation *in vitro*, cell culture plates were coated with the indicated concentration of anti-CD3ε antibodies (BioXcell; clone 145-2C11; BE0001-1 for mouse or Bio-X-Cell; clone OKT3; BE0001-2 for human) in PBS. Anti-CD28 antibodies (BioXcell; clone 37.51; BE0015-1) were added at a final concentration of 1 μg/mL soluble to the culture. For flow cytometric analysis of cytokine production, cells were stimulated for 4 h with 50 ng/mL phorbol-12,13-dibutyrate (Sigma, P1269) and 500 ng/mL ionomycin (Sigma, I0634) and treated with brefeldin A and monensin (1:1000 and 1:1500; BD Biosciences, 555029 and 554724).

#### RNA extraction, cDNA synthesis and quantitative real time (qRT-)PCR

RNA was extracted using the RNeasy Mini Kit (Qiagen,Cat.no.74106) according to the manufacturer’s protocol. For cDNA synthesis, Omniscript RT Kit (Qiagen, Cat.no.205111), Oligo-dT 15 primer (Promega, Cat.no.C1101) and RNase inhibitor (Promega, Cat.no.N2111) were used according to the manufacturer’s protocol. Real-time PCR was performed using Luna® Universal qPCR Master Mix and TaqMan probes for the relevant genes on a 7500 FAST Real-Time PCR system.

#### Whole cell lysis

Cells were collected, washed once with cold PBS and lysed for 30 min on ice in 30 μL lysis buffer (5 mM Na_4_P_2_0_7_, 5 mM NaF, 1 mM Na_3_VO_4_, 5 mM EDTA, 150 mM NaCl, 50 mM Tris [pH 7.3], 1% NP-40, aprotinin and leupeptin [50 μg/mL each]). After centrifugation (16000 × g/15 min/4°C), the supernatant containing cellular proteins was mixed with 5× SDS-loading buffer (Thermo Fisher Scientific; #39000) and stored at −20°C or directly applied to a gel.

#### Nuclear extracts

Cells were harvested, washed once with cold PBS (1 min, 9,300 × g, 4°C), resuspended in 300 μL of Buffer A (10 mM HEPES pH 7.9, 10 mM KCl, 0.1 mM EDTA, 0.1 mM EGTA, 1 mM DTT, 0.5 mM PMSF) and kept on ice for 15 min. 20 μL 10% NP-40 were added, the pellet was vortexed and washed twice with 300 μL of Buffer A (5 min, 2,300 × g, 4°C). The nuclear pellet was taken up in 30 μL of Buffer C (20 mM HEPES pH 7.9, 0.4 M NaCl, 1 mM EDTA, 1 mM EGTA, 1 mM DTT, 1 mM PMSF) and put on a shaker for 30 min at 4°C. After centrifugation (10 min, 13,400 × g, 4°C), supernatants were taken and stored at −80°C. Protein concentrations were determined by Bradford assay.

#### SDS PAGE and immunoblot

Extracts were heated with reducing sample buffer (Thermo Fisher Scientific) to 95°C for 5 min before loading on a 4–12% Bis-Tris gel (Thermo Fisher Scientific; #10472322) in a MOPS-based buffer (50 mM MOPS, 50 mM Tris, 3.5 mM SDS, 1 M EDTA, in a.d.; pH = 7.7). Proteins were transferred to a methanol-activated PVDF membrane (Immobilon-P, Merck; IPVH00010) using a semi-dry method (transfer buffer: 25 mM bicine, 25 mM BIS-TRIS, 1.025 mM EDTA, in a.d.). Unspecific binding sites were blocked by 1 h incubation with blocking solution (5% non-fat milk powder in TBST: 5 mM Tris, 75 mM NaCl, 0.5% v/v Tween, in a.d., pH = 8.0). Membranes were incubated on a shaker overnight at 4°C with primary antibodies (1:1000 NLK (D9X3C), Cell Signaling Cat#94350; 1:2000 β-Actin (C-4), SantaCruz Cat#sc-47778; 1:1000 NFATc1 (7A6), SantaCruz Cat# sc-7294; 1:1000 Lamin B1, Abcam Cat#ab16048). On the next day, the membrane was washed with TBST and incubated for 1 h at RT with horseradish peroxidase (HRP)-coupled secondary antibodies (1:5000). After washing with TBST, proteins of interest were detected by SuperSignal West Pico Chemiluminescent Substrate (Thermo Scientific, #34580) on film. Densitometric analysis was performed using ImageJ.

#### Gel mobility shift assay

Single-stranded oligonucleotides were synthesized by Eurofins MWG Operon and annealed. CD8^+^ T cells from wt or NLK^fl/fl^ mice were lysed and nuclear extracts were prepared. Protein extracts (4 μg) were incubated at 4°C with ^32^P end-labeled, double-stranded oligonucleotide probe (NFAT: GCCCAAAGAGGAAAATTTGTTTCATACAG or AP-1: CGCTTGATGACTCAGCCGGAA) in binding buffer (10 mM Tris pH 7.5, 50 mM NaCl, 1 mM DTT, 1 mM EDTA, 5% glycerol, 0.1% BSA, 0.3 μg poly dI-dC). Band shifts were resolved on a 5% polyacrylamide gel. Where indicated, 1 μg of supershift antibody anti-NFATc1 (7A6) was added.

#### Cytokine measurements

Cell culture supernatants were analyzed using Luminex xMAP technology (Bio-Rad) with the Bio-Plex Pro Mouse Chemokine IL-2 Set (12002439), Bio-Plex Pro Mouse Chemokine IFN-γ Set (12002438), Bio-Plex Pro Human Cytokine IL-2 Set (171-B5003M) and the Bio-Plex Pro Human Cytokine IFN-γ Set (171-B5019M), and cytokine production on a per cell level was analyzed by flow cytometry (see separate section).

#### [^3^H]-thymidine incorporation

On day 1 of culture, 50 μL supplemented RPMI 1640 containing 1 μCi [^3^H]-thymidine (PerkinElmer) were added. After a 16-h pulse, cells were harvested and [^3^H]-thymidine incorporation was measured with a 1450 Microbeta Trilux Liquid Scintillation and Luminescence Counter (Wallac).

#### Repeated antigen stimulation *in vitro*

The repeated stimulation assay was adapted from Zhao and colleagues.^26^ In brief, CD8^+^ T cells from spleens and lymph nodes of OT-I mice were cultured in complete RPMI supplemented with 10% fetal calf serum, 100 U/mL penicillin, 100 μg/mL streptomycin, 2 mM L-glutamine, 100 nM sodium pyruvate, 100 nM non-essential amino acids, 25 mM HEPES and 50 μM β-mercaptoethanol. Additionally, cells were supplemented with 5 ng/mL IL-15 (Biolegend, 570302) and 5 ng/mL IL-7 (Biolegend, 581902) with or without 10 ng/mL OVA(257–264) SIINFEKL peptide (Eurogentec, AS-60193–5).

For single peptide stimulation, cells were kept in culture with 10 ng/mL OVA(257–264) SIINFEKL peptide for 48 h. The cells were then washed twice with complete media to remove the peptide and afterward cultured in complete media with cytokines until day 5.

For repeat peptide stimulation, 10 ng/mL OVA(257–264) SIINFEKL peptide was added every day. To ensure comparable culture conditions, the cells were also washed twice on day 2. On day 5, cells were harvested for analysis.

#### Flow cytometric analysis

Depending on the experiment, the following steps were performed:

• CFSE labeling: Cells were washed with HBSS, incubated for 5 min at 37°C in 2.5 μM CFSE solution (in HBSS) and labeling was stopped by adding FCS. Cells were resuspended in medium and stimulated for three days before surface staining and measurement.
• MC38-Tetramer staining: According to the manufacturer's instructions, the MC38 tetramer was spun down at 3000 g for 5 min and only the supernatant was used for staining. Per sample, 5 μL MC38 tetramer (=0.25 μg tetramer) and 45 μL PBS/2% FCS were added to the 50 μL residual cell pellet and mixed by vortexing. After 20 min incubation (37°C and 5% CO2) the cells were washed once with PBS.
• Live/dead stain:

For mouse samples, 50 μL of fixable viability stain FVS440UV (BD HorizonTM; Cat. No. 566332) (1:125 in PBS) were added to 50 μL cell suspension. After 10 to 15 min incubation at room temperature and protected from light, the cells were washed two times with FACS buffer (PBS, 0.5% BSA, 2 mM EDTA).

For human samples, BD Horizon Fixable Viability Stain 780 (BD 565388), diluted 1:2000 in HBSS, was used instead.

• Fc receptor block (Biolegend; Cat. No. 101320, clone: 93) was added to the cells to avoid unspecific binding (final dilution 1:100 in FACS buffer). After 5 min incubation at 4°C, 50 μL 2× concentrated surface antibody staining mix was added to 50 μL cell suspension. The 2× antibody mix was diluted in FACS buffer, optionally containing 20% Brilliant Stain Buffer Plus (BD HorizonTM; Cat. No. 566385) to prevent fluorescent dye interactions, causing subsequent staining artifacts. Cells were incubated for 20 min at 4°C protected from light and washed twice with FACS buffer.
• Intracellular staining: cells were fixed and permeabilized (Biolegend 421002 and 420801 for cytokines; eBioscience 00–5523-00 for transcription factors). Intracellular antigen antibody-mix was applied in permeabilization buffer and later washed two times with FACS buffer.
• 7AAD addition (1:100) directly before measurement
• Measurement was performed using a FACS Calibur (BD), a FACS Canto II (4-2-2 configuration, BD) or a 5-laser Cytek®Aurora spectral cytometer.
• Data analysis was performed using FlowJo (version 10.8.1 and 10.10.1).

#### *In vitro* T_reg_ differentiation

Naive CD4^+^ cells (positively selected from presorted CD4^+^ cells via CD62L MicroBeads, Miltenyi) were treated with 2 μg/mL anti-CD3 (plate-bound), 5 ng/mL TGFβ, 20 ng/mL IL-2, 5 μg/mL anti-IL-12, 5 μg/mL anti-IFNγ, 5 μg/mL anti-IL-4 in supplemented RPMI 1640 for three days. Subsequently, cells were analyzed for Foxp3 expression.

#### *In vitro* T_reg_ suppression assay

CD25^+^CD4^+^ and CD25^−^CD4^+^ T cells were isolated from erythrocyte-depleted cell suspensions of spleens and lymph nodes of NLK^+/+^ and NLK^fl/fl^ mice using the CD4^+^ T cell isolation kit II followed by anti-CD25-PE and anti-PE MicroBeads (all Miltenyi Biotec) according to the manufacturer’s instructions. Sorted CD25^−^CD4^+^ T cells were labeled with 2.5 μM CFSE for 4 min at 37°C; labeling was stopped by the addition of FCS. T-cell-depleted splenocytes (using CD4 and CD8a MicroBeads; Miltenyi Biotec) were treated for 45 min with 50 μg/mL mitomycin C (AppliChem) and then washed for three times with supplemented RPMI 1640. These cells were used as antigen-presenting cells (APCs). To induce proliferation, 0.5 μg/mL of anti-CD3 (clone 2C-11) was added. 1 × 10^5^ CFSE-labeled CD25^−^CD4^+^ responder T cells were cultured with 1 × 10^5^ APCs in 96-well U-bottom tissue culture plates (Falcon). CD25^+^CD4^+^ suppressor T cells were added at the ratios 1 + 1, 4 + 1 and 9 + 1 (responder + suppressor cells). On day 3 of co-culture, proliferation (based on CFSE dilution) was analyzed by flow cytometry; 7-AAD was added to exclude dead cells from the analysis.

#### Bulk RNAseq

*For in vitro repeated stimulation assay*: Total RNA was isolated using the RNeasy Micro kit (Qiagen) according to manufacturer’s instructions. RNA was eluted in the kits elution buffer and stored at −80°C before quality control (QC). Purified total-RNA was submitted for transcriptome analysis at the Medical University Innsbruck MultiOmics Sequencing Core for the purpose of gene-expression profiling, using the QuantSeq 3′ mRNA-Seq Library Prep method (Lexogen GmbH, Vienna Biocenter). Quality-validated, barcoded libraries were multiplexed and sequenced using Illumina NovaSeq technology (GeneWiz/Azenta, Leipzig, Germany).

The nf-core/rnaseq (v3.14.0) pipeline^51^ was used to align the raw reads to the mouse genome (GRCm39) with STAR and to assess the read counts on the gene models from GENCODE version M31 with Salmon. The pipeline was run using default parameters, but to accommodate the QuantSeq 3′-mRNA library data, the option ‘--extra_star_align_args’ was modified as recommended in the documentation and ‘--noLengthCorrection’ was used for gene quantification with Salmon.^52^ DESeq2 (v1.42.1) was employed to identify differentially expressed genes between wild-type single stimulated and wild-type repeated stimulated (exhausted) T cells, applying a fold change threshold of 2 and a false discovery rate (FDR) of 0.1 after Independent Hypothesis Weighting (IHW).^53^

*For CD8*^*+*^ *TILs*: Single-cell suspensions were generated as described above. CD8^+^ cells were isolated via positive selection using CD8a microbeads (Miltenyi Biotec; 130–117-044). This enriched fraction was stained with CD8-APC and DAPI and then sorted for viable CD8^+^ cells using a FACSAria cell sorter.

RNA was isolated using the RNeasy Micro kit (Qiagen) according to manufacturer’s instructions. Purified total RNA was submitted to Novogene. Non-strand specific library was prepared using NGS RNA Library Prep Set (Novogene; PT042). Briefly, mRNA was selectively captured from total RNA using poly-T oligo-attached magnetic beads. After fragmentation, cDNA synthesis was carried out in two steps: first-strand synthesis with random hexamer primers, followed by second-strand synthesis. The library was then constructed through a series of steps, including end repair, A-tailing, adapter ligation, size selection, amplification, and purification. To ensure quality, Qubit and real-time PCR were used for quantification, while a bioanalyzer assessed size distribution. Subsequently, libraries were subjected to Illumina Sequencing. Raw data of fastq format were cleaned through fastp software and subsequently mapped to the reference genome (Mus musculus, GRCm39/mm39) using HISTAT2 (2.2.1) (all at Novogene).

For improved signal to noise ratio and thus also statistical power, raw count table was pre-filtered for protein-coding genes only with at least 5 total counts in half of the samples. Differential gene expression analysis was performed using the DESeq2 package (v1.42.1). Normalization was performed using the median of ratios method. Wald test together with Benjamini-Hochberg (BH) correction were applied to identify differentially expressed genes (DEGs) with padj ≤0.05 and log2 fold change ≥1 being defined as thresholds. R-log transformed data were used for visualization of variance across samples by principal component analysis (PCA) and expression pattern of selected genes showing robust Z-scores in heatmaps. The latter were calculated for each gene by subtracting the median and dividing by the interquartile range. For Gene Set Enrichment Analysis (GSEA), data were mapped from ENSEMBL gene IDs to Entrez IDs using the org.Mm.e.g.,.db annotation database (v4.3.1) and ranked by LFC in descending order. KEGG (Kyoto Encyclopedia of Genes and Genomes) pathway enrichment was performed with clusterProfilergseKEGG (v4.8.1). Selected significantly enriched pathways were visualized using enrichplotgseaplot2 (v1.20.2).

#### Human T cell isolation, culture and cell counting

Peripheral blood mononuclear cells (PBMCs) were obtained from leukocyte reduction cones (LRCs) collected from healthy donors by density gradient centrifugation. Briefly, LRCs were rinsed with HBSS (PAN Biotech, P04 34500) supplemented with 2 mM EDTA, and the cell suspension was carefully layered onto Lymphopure solution (BioLegend, 426202) prior to centrifugation at 800 rcf for 30 min at room temperature (RT) without brake. The PBMC layer was recovered, washed with HBSS containing 2 mM EDTA, and pan-T cells were subsequently enriched by negative magnetic selection using the MojoSort Human CD3 Isolation Kit (BioLegend, 480131) together with LS columns and a QuadroMACS separator (Miltenyi Biotec, 130 042 401 and 130 091 051), following the manufacturers’ instructions.

Purified T cells were maintained in complete RPMI 1640 (cRPMI) consisting of RPMI 1640 (PAN Biotech, P04 17500) supplemented with 10% heat-inactivated FBS (Biowest, S1810 500), 100 U/mL penicillin and 100 μg/mL streptomycin (PAN Biotech, P06 07100), 2 mM L-glutamine (PAN Biotech, P04 80050), 1 mM sodium pyruvate (Sigma, S8636), and 1× non-essential amino acids (Sigma, M7145). Cell concentration and viability were determined using a LUNA automated cell counter (Logos Biosystems) with acridine orange/propidium iodide staining (Biocat, F23001 LG).

#### Lentiviral NLK OE and CAR expression

Stable *NLK* overexpression (OE) was generated using EF1α-driven HA-tagged *NLK* or control construct encoding β-galactosidase (aa 2–83) linked via an IRES to puromycin resistance (VectorBuilder, VB241126-1331hgg and VB260129-1174nxw). CAR expression employed a second-generation EGFR-specific construct comprising a Cetuximab-derived scFv, CD8 hinge and transmembrane domains, CD28 costimulatory domain, and CD3ζ signaling domain (VectorBuilder, VB241014-1216jtq).

Stable integration and enrichment of modified cells were performed as established and recently described by Koutník et al.^54^

Briefly, lentiviral particles were generated in HEK293T cells cultured in antibiotic-free medium by transient transfection with third-generation lentiviral packaging plasmids (Creative Biolabs, CART 027CL; 0.9 pmol pHELP1, 1.5 pmol pHELP2, and 1.3 pmol pHELP3) together with 2.25 pmol of the transfer plasmid. Plasmid DNA was pre-diluted in Opti-MEM (Thermo Fisher Scientific, 31985062) and complexed with Lipofectamine 2000 (Thermo Fisher Scientific, 11668019) according to the manufacturer’s instructions before being added dropwise to the cells. The medium was replaced 6–18 h after transfection. Viral supernatants were collected up to 60 h post-transfection and concentrated using Lenti-X Concentrator (Takara, 631232).

For stable genetic modification, CD3^+^ T cells were activated on day 0 using αCD3/αCD28 Dynabeads (Thermo Fisher, 11132D). After 24 h (day 1), cells were co-transduced in the presence of polybrene (8 μg/mL; Sigma, TR 1003 G). Transduced cells were selected with puromycin (2.5 μg/mL; Sigma, P8833) from days 3–5, followed by Dynabead removal and subsequent expansion. On day 10, CAR^+^ cells were enriched by recombinant EGFR-based magnetic-activated cell sorting (MACS) using MS columns and an OctoMACS Separator (Miltenyi, 130 042 201 and 130 042 109) based on the manufacturer’s instructions. All enrichment steps were performed at 4°C in CAR buffer (PBS supplemented with 2 mM EDTA and 2% BSA). Cells were blocked for 15 min, labeled with biotinylated EGFR (0.5 μg/mL; Creative Biolabs, CARD LX025) and anti-biotin microbeads (2.5 μL per 10^6^ cells; Miltenyi, 130 090 485) for 20 min, and subjected to magnetic separation. The positive fraction was collected, and enrichment purity was assessed by flow cytometry. Cells were subsequently expanded until day 13.

Throughout the culture period, cells were maintained in cRPMI supplemented with 10 ng/mL IL-2 (BioLegend, 589102). Medium was refreshed every 2–3 days, and cell density was adjusted to 0.5–1 × 10^6^ cells/mL.

#### CAR T – A549 cell co-culture

A549 *B2M*^−/−^ GFP^+^, stably transduced with pLVX-EGFP-IRES-puro (gift from R. Sobol, Addgene #128652, described by Fang et al.^49^) target cells (5 × 10^5^) were seeded into 96-well plates 12–16 h before addition of effector cells at a 4:1 effector-to-target ratio (200 μL cRPMI). Target cell killing was monitored by live-cell imaging using an Incucyte SX5 system (Sartorius). Fluorescent images were acquired at 10× magnification (4 fields per well, every 2 h). Fluorescence was quantified using Incucyte 2023A software as total integrated intensity, using predefined segmentation parameters (Surface Fit with threshold of 1 calibrated unit; edge split on).

For long-term challenge with tumor cells, every 2–3 days 100 μL of supernatant were replaced with fresh medium containing tumor cells for up to 10 days before analysis by flow cytometry.

### Quantification and statistical analysis

Results are presented and statistical significance was assessed as indicated in the respective figure legends; *p* < 0.05 was considered significant. ∗*p* < 0.05, ∗∗*p* < 0.01, ∗∗∗*p* < 0.001, ∗∗∗∗*p* < 0.0001. Unless otherwise stated, murine data are presented as mean ± SEM and human donor data as matched values, and differences were analyzed using Student’s *t* test, paired where appropriate. n-numbers represent the number of animals. Statistical analyses and graphical representations were conducted using GraphPad Prism version 10.2.3 (GraphPad Software, Inc.).Tumor growth and CAR T cell killing curves were analyzed by two-way ANOVA with Šidák’s multiple-comparison test, with genotype × time interactions and comparisons at the final time point considered relevant. Survival was analyzed using Cox regression models adjusted for experiment number, reporting median survival, hazard ratio, and *p* value. RNA-seq data were analyzed using DESeq2, employing the Wald test with Holm–Bonferroni correction for multiple testing.
