# Supplementary material for: Nemo-like kinase limits T cell activation while preserving anti-tumor responses
Source: iScience. 2026 Nov 2;29(11):118070. doi: 10.1016/j.isci.2026.118070 (PMC13755957; doi:10.1016/j.isci.2026.118070)
Supplement: Supplementary file 1 [file mmc1.pdf]

iScience, Volume *29*

Supplemental information

Nemo-like kinase limits T cell activation while preserving anti-tumor responses

Sebastian Peer, Jiří Koutník, Nikolaus Thuille, Kerstin Siegmund, Victoria Klepsch, Maria Pfeiffer, Dominik Humer, Patrick Bantsich, William J. Olson, Karin Albrecht-Schgoer, Gottfried Baier, and Thomas Gruber

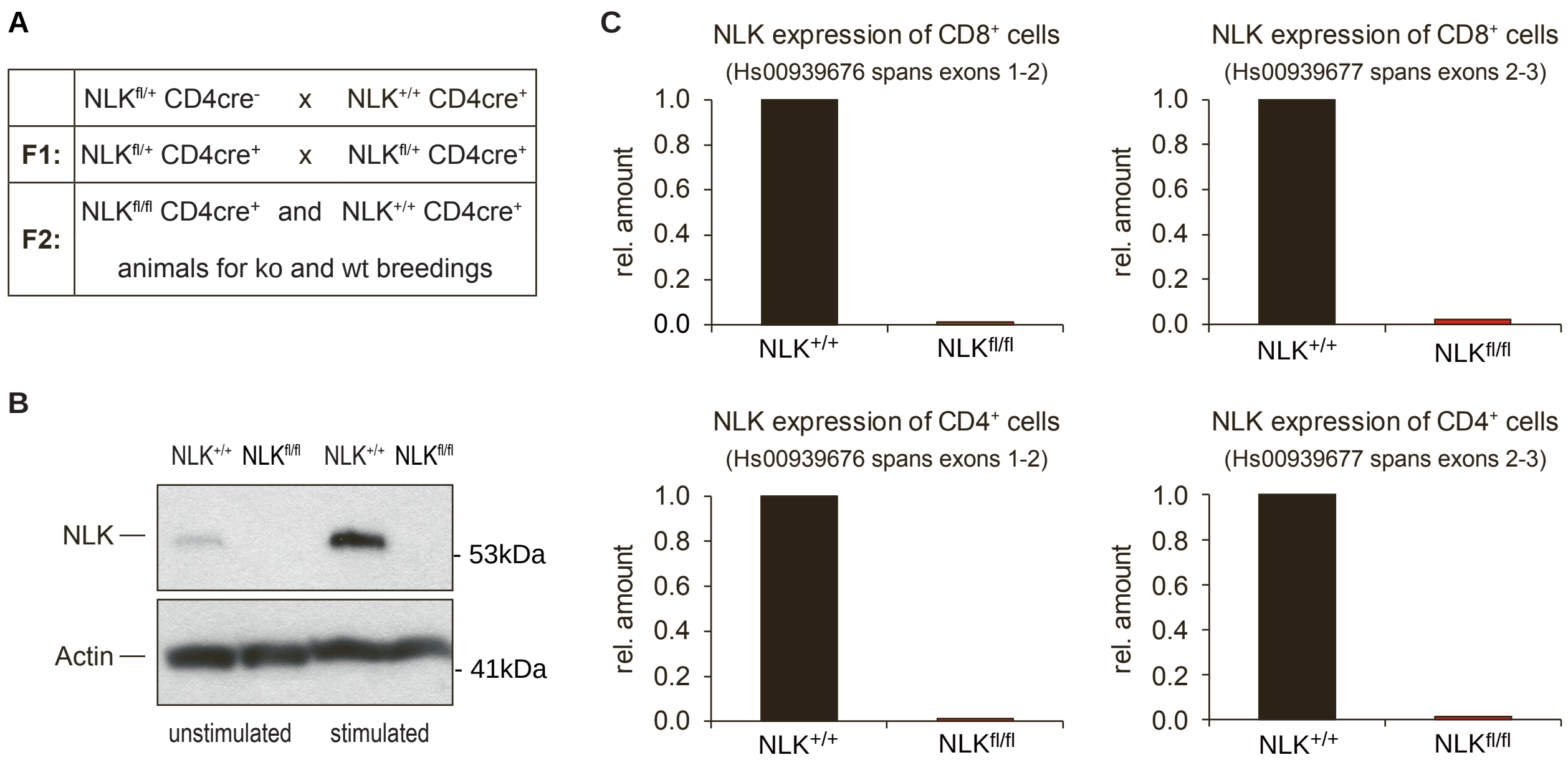

Supplementary Figure 1. Generation of T cell-specific NLK knockout mice.
(A) Breeding strategy. **(B)** Representative western blot of $CD3^+$ cells left unstimulated or stimulated for 19 hours with 5 µg/ml plate-bound anti-CD3 and 1 µg/ml soluble anti-CD28. NLK and β-Actin in whole cell lysates are shown. **(C)** NLK mRNA expression in $CD8^+$(upper panel) and $CD4^+$ cells (lower panel), stimulated as in **(B)**. Expression was quantified with qRT-PCR using two different TaqMan probes (Hs00939676 and Hs00939677) with GAPDH as a control gene. NLK expression in wild-type cells was set to 1.

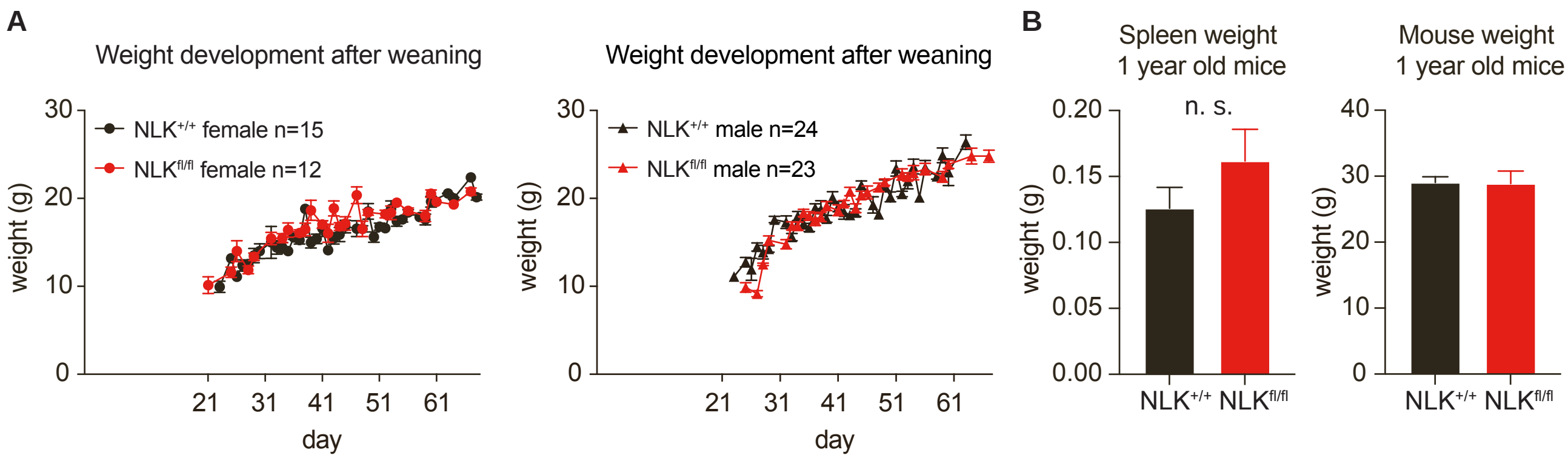

Supplementary Figure 2. Mouse and spleen weights. (A) Mouse weight development after weaning. (**B)** Spleen (n=12-13) and mouse (n=7-11) weight of one year old mice. Data are represented as mean +/- SEM. Unpaired t-test; n. s. = not significant.

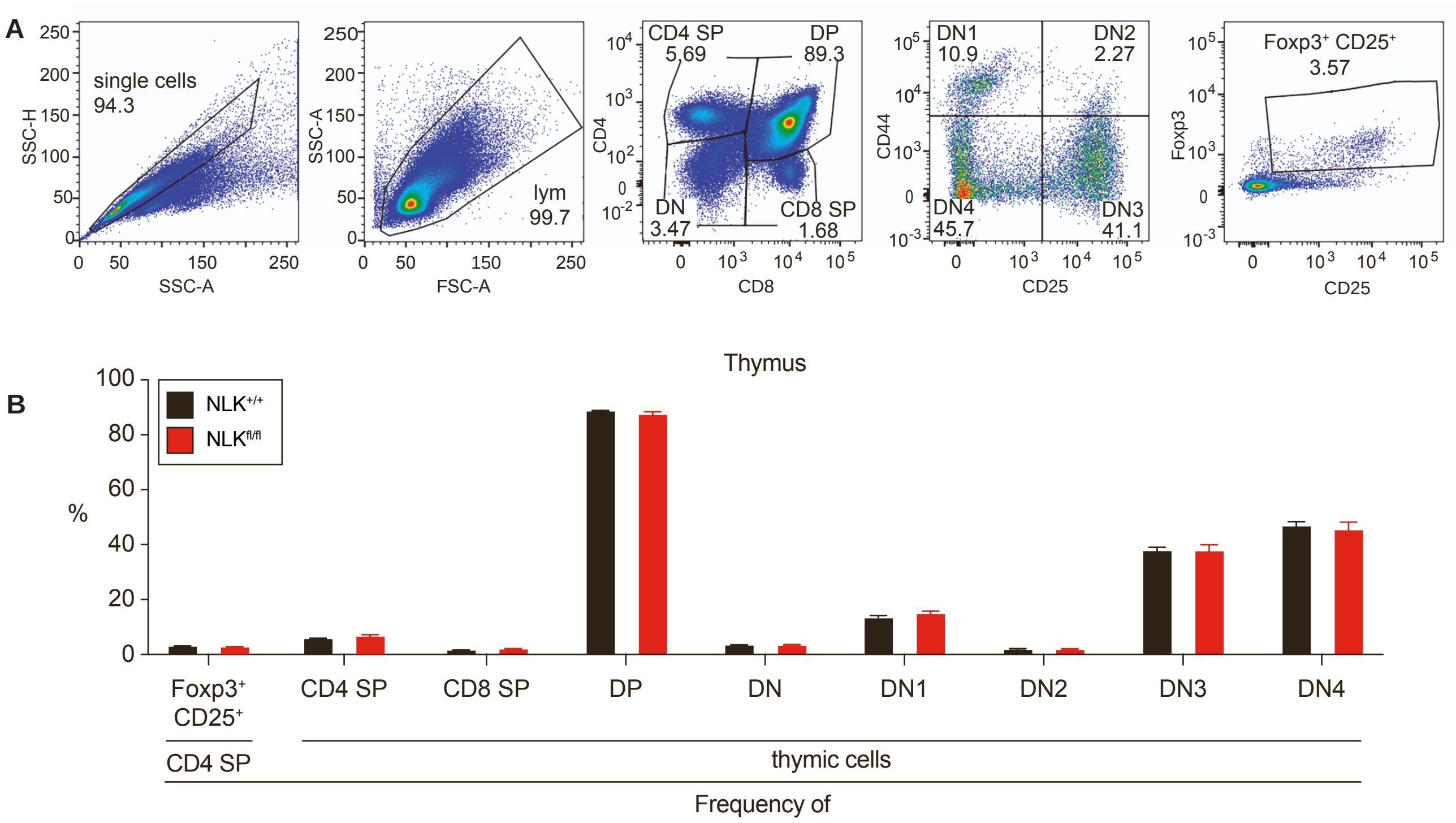

Supplementary Figure 3. Thymocyte development. (A) Representative gating strategy of thymic cell suspensions. **(B)** Percentages of subsets from two independent experiments (SP=single positive, DN = double negative; DN1: $CD44^{+}CD25^{-}$, DN2: $CD44^{+}CD25^{+}$, DN3: $CD44^{-}CD25^{+}$, DN4: $CD44^{-}CD25^{-}$); n=6 mice. Data are represented as mean +/- SEM.

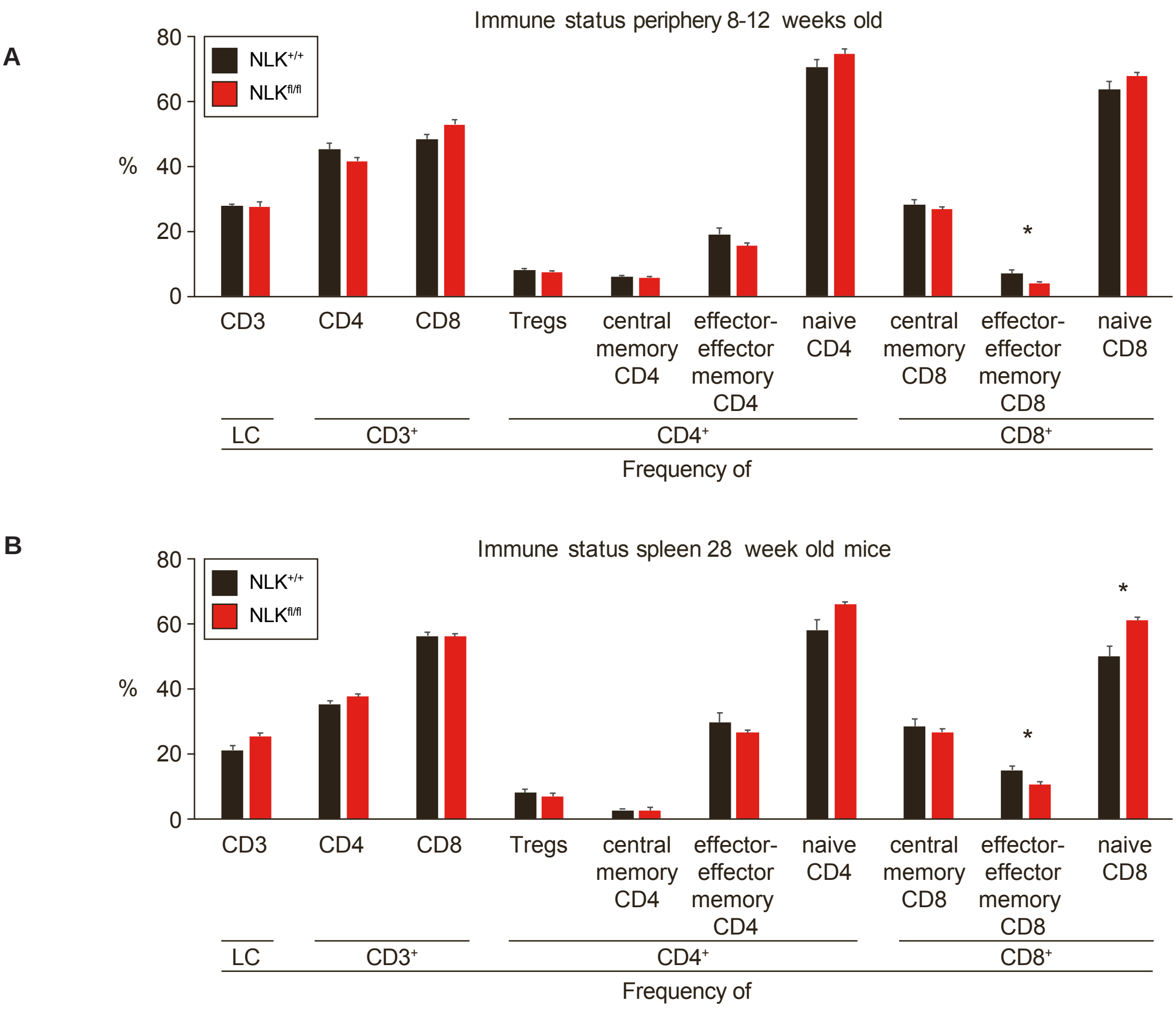

Supplementary Figure 4. T cell subsets in peripheral lymphoid organs. Flow cytometric analysis of peripheral lymphoid organs from **(A)** 8-12 week old mice (spleen & lymph nodes combined; n=6 from two independent experiments) and **(B)** 28 week old mice (spleen; n=6 from two independent experiments). T_{regs}: $CD3^+CD4^+CD25^+Foxp3^+$, central memory: $CD3^+CD4^+CD44^+CD62L^+$ or $CD3^+CD8^+CD44^+CD62L^+$, effector memory: $CD3^+CD4^+CD44^+$ $CD62L^-$ or $CD3^+CD8^+CD44^+CD62L^-$, naive: $CD3^+CD4^+CD44^-CD62L^+$ or $CD3^+CD8^+CD44^-$ $CD62L^+$. Data are represented as mean +/- SEM. Unpaired t-test; *$p < 0.05$

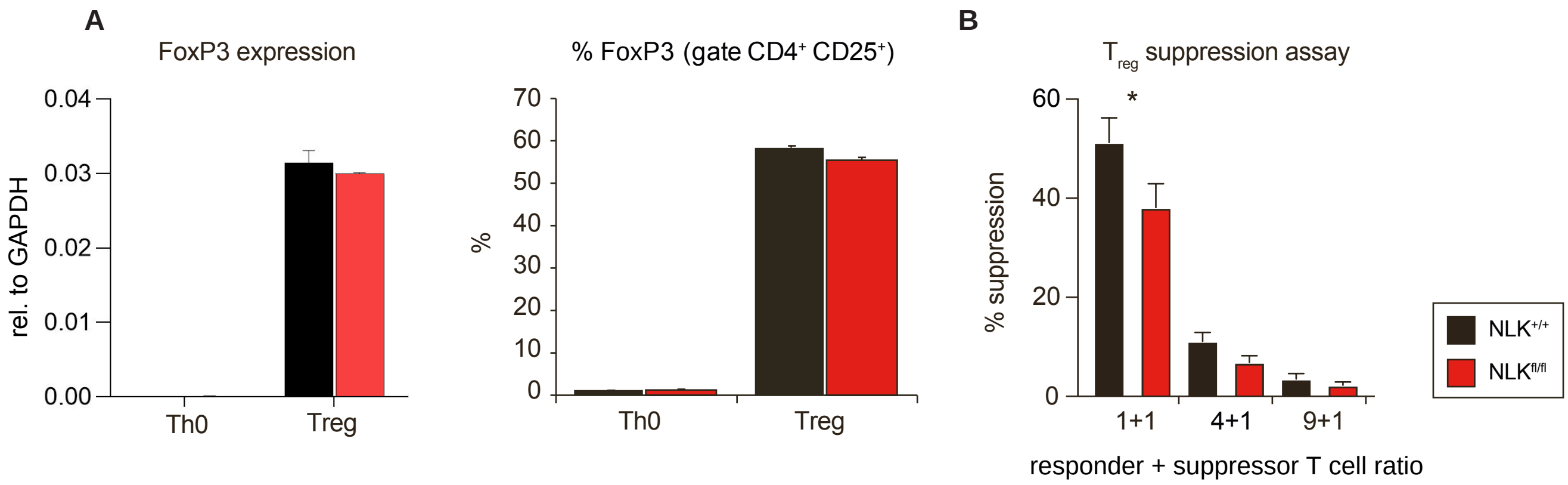

Supplementary Figure 5. ***In vitro*** $\mathbf{T_{reg}}$ **differentiation and function**. **(A)** qRT-PCR (left) and flow cytometric analysis (right) of *in vitro* differentiated $NLK^{+/+}$ and $NLK^{-/-}$ $CD4^{+}$ regulatory T cells; n=2. (**B)** Wild-type and NLK-deficient $CD25^{+}CD4^{+}$ suppressor T cells were co-cultured with wild-type $CD25^{-}CD4^{+}$ responder T cells at indicated ratios together with antigen-presenting cells and anti-CD3 for three days. Proliferation was analyzed by flow cytometry based on CFSE dilution. Percent suppression is shown (responder proliferation without the addition of suppressor cells is set to 0 % suppression); n=6 from two experiments. Data are represented as mean +/- SEM. Unpaired t-test; *p < 0.05.

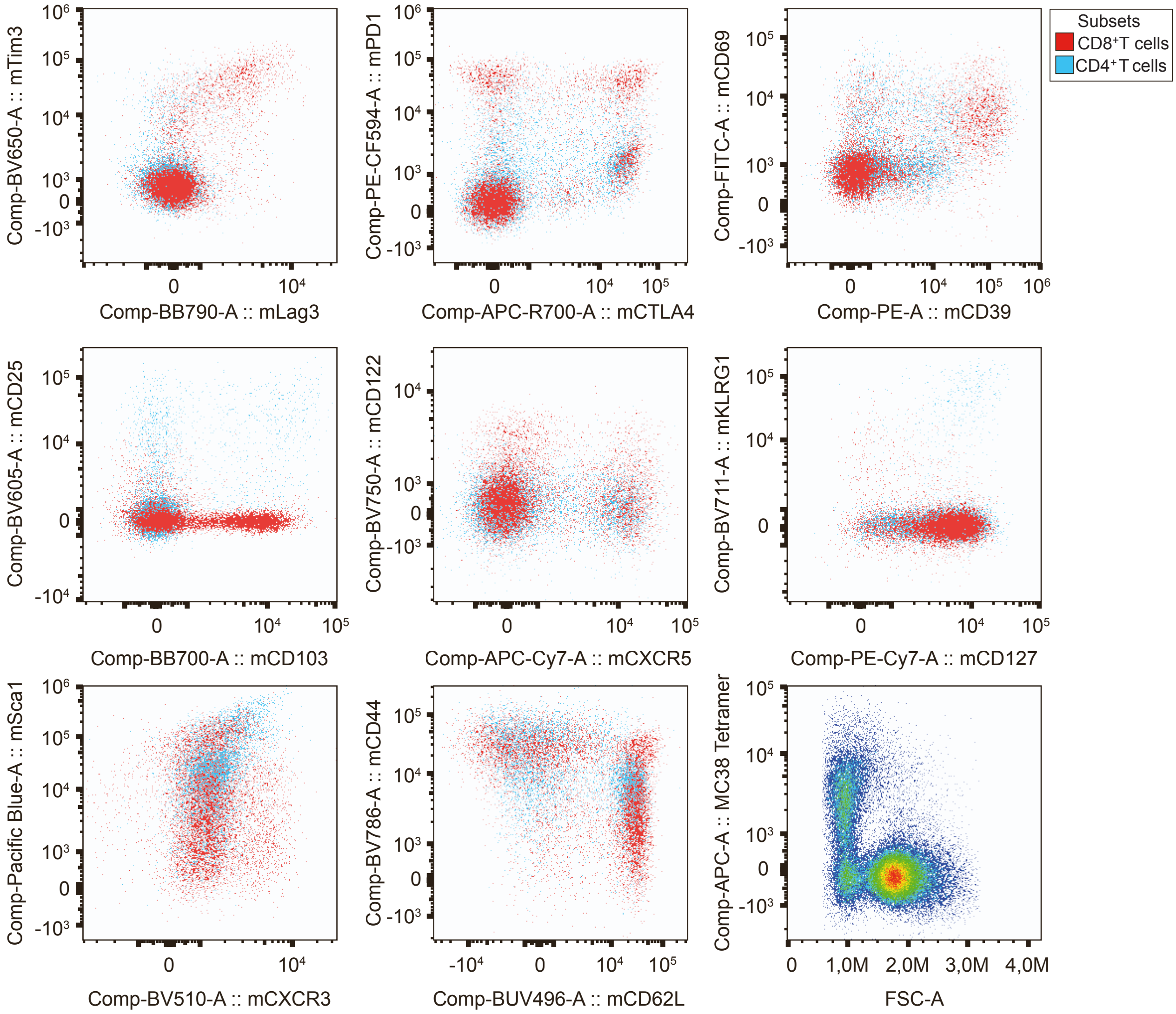

Supplementary Figure 6. Exemplary marker expression for FlowSOM analysis of Fig. 5C-E. MC38 tetramer staining is shown only for CD8⁺ cells.

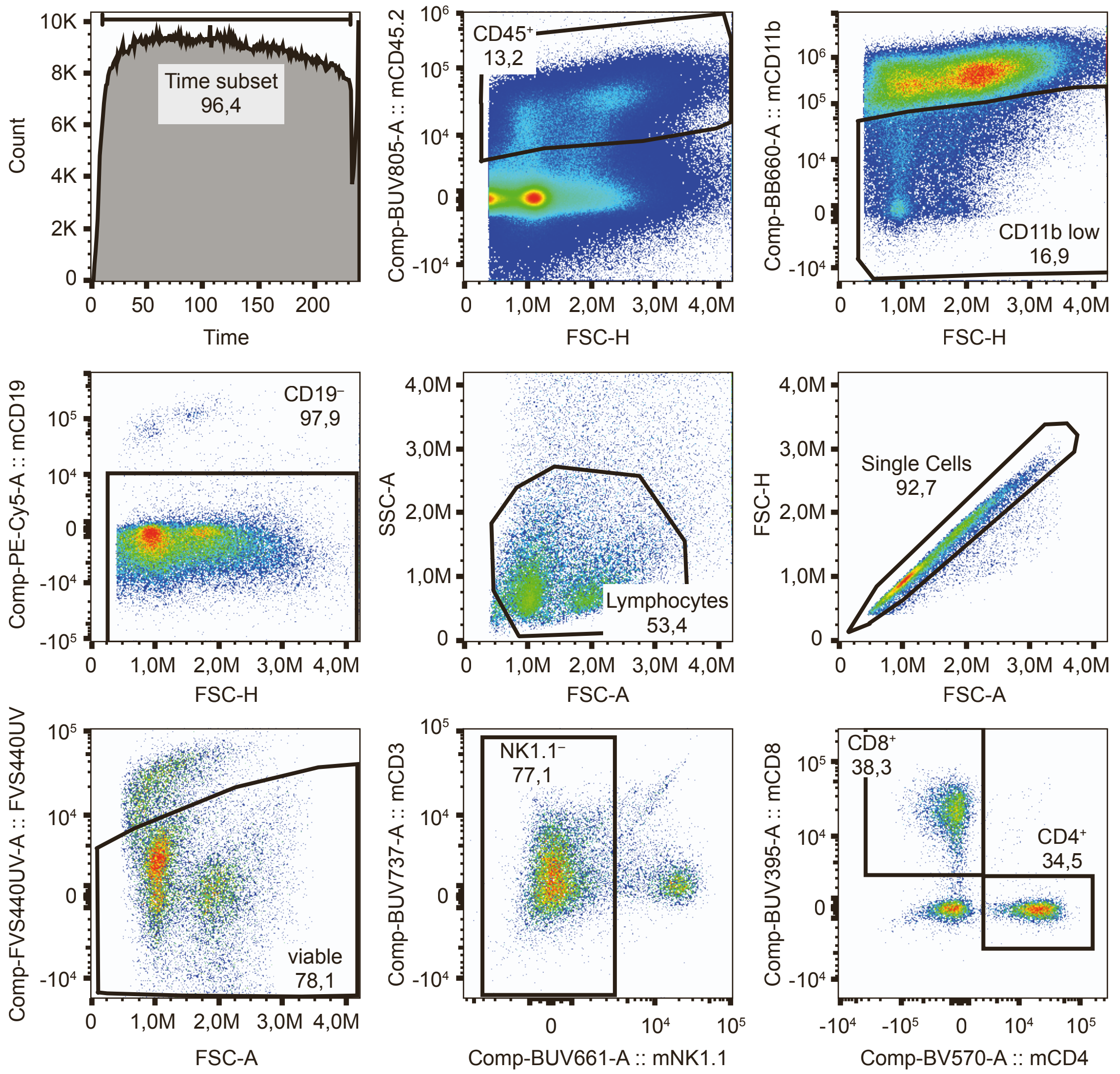

Supplementary Figure 7. Gating strategy for FlowSOM analysis of Fig. 5C-E.

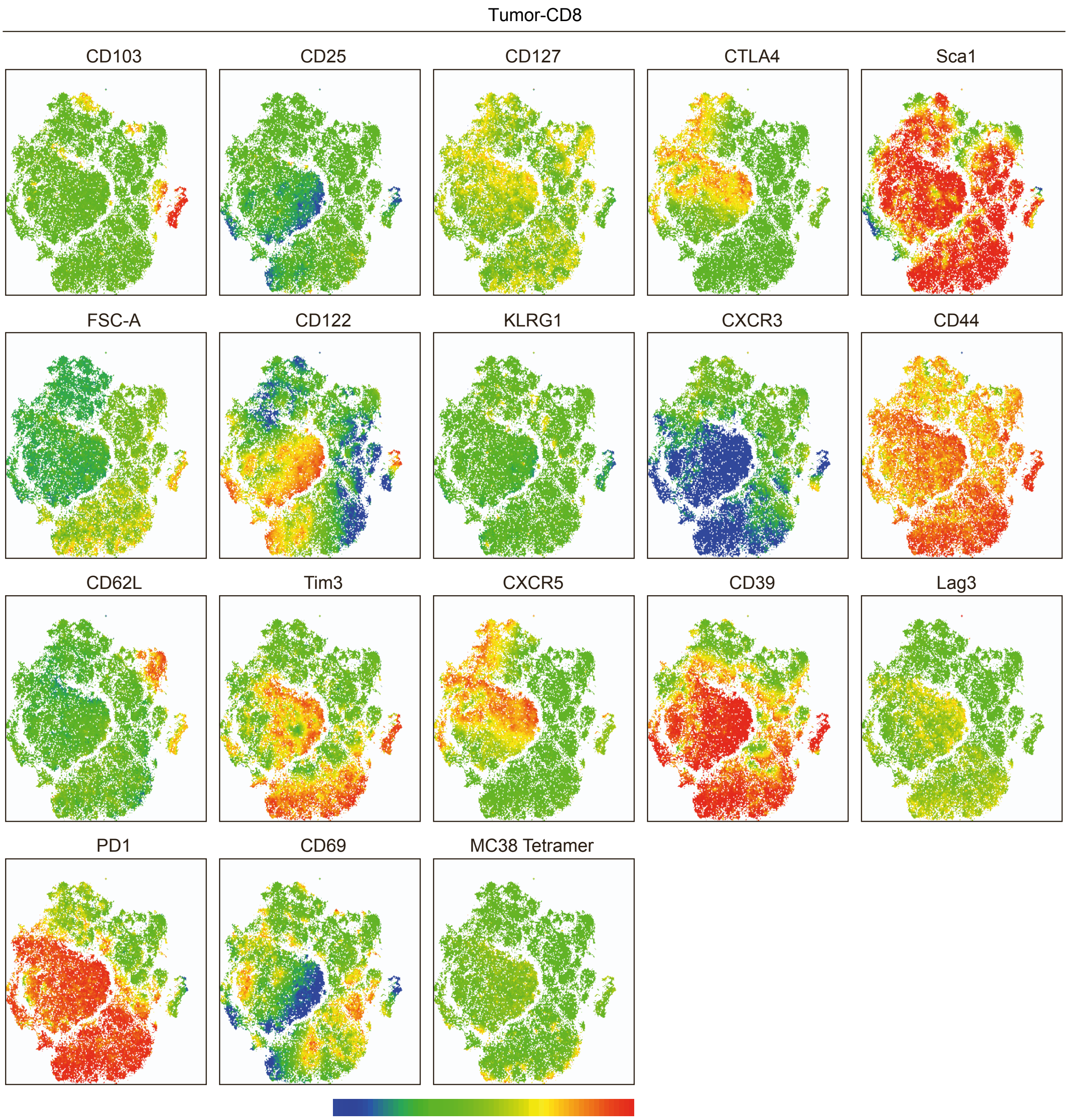

Supplementary Figure 8. Heatmap showing expression intensity of the used markers on the t-SNE plot of Fig. 5C.

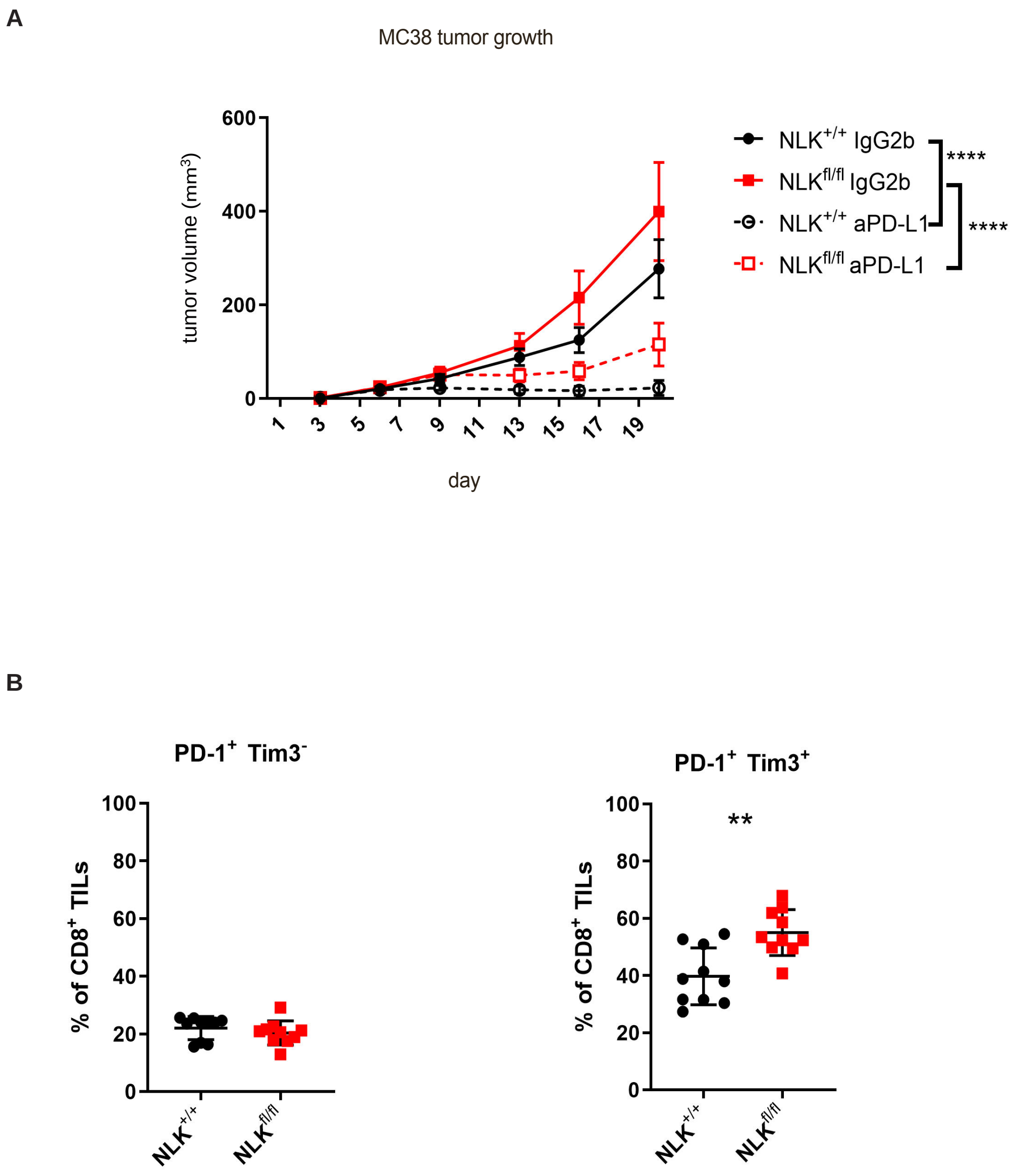

Supplementary Figure 9. Blockade of PD-L1 partially restores antitumor activity of NLK-deficient T cells. (A) 500.000 MC38 colon carcinoma cells were injected subcutaneously and tumor volume was monitored. 250 µg anti-PD-L1 or IgG2b control was administered i. p. twice a week. Mean and SEM are shown, n=8-9. **(B)** Flow cytometric analyses were performed three weeks after MC38 injection. Staining of PD-1 and Tim-3 on intratumoral CD8$^+$ T cells is shown. Two-way ANOVA (A), unpaired t-test (B); **p < 0.01,****p< 0.0001.

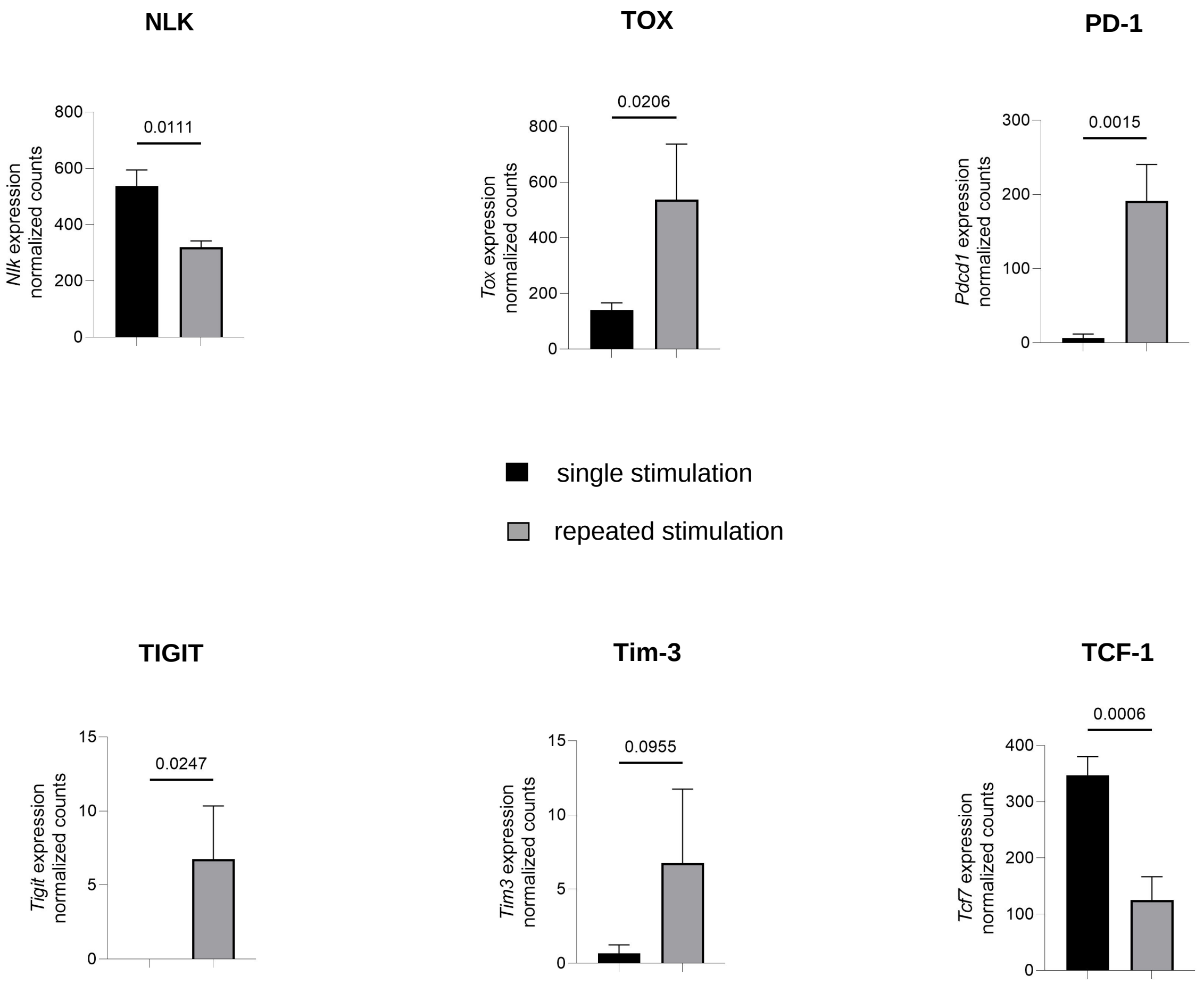

Supplementary Figure 10. NLK expression is downregulated during repeated stimulation. $CD8^+$ T cells of OT-I mice were stimulated with SIINFEKL peptide once ("single stimulation") or daily for five days ("repeated stimulation"). Cells were harvested for bulk RNA sequencing and expression of the indicated genes is shown (protein names are also denoted). The p-values of unpaired t-tests are given in the respective diagrams, n=4.

Surface marker/cell type	NLK$^{+/+}$	NLK$^{fl/fl}$	p
CD45^{+}	15,569 +/- 1,536	14,087 +/- 2,211	0.59
CD8^{+}	482 +/- 97	487 +/- 101	0.97
CD4^{+}	378 +/- 103	224 +/- 42	0.18
B cells	58 +/- 10	61 +/- 13	0.86
NKT cells	56 +/- 9	76 +/- 25	0.48
NK cells	216 +/- 38	130 +/- 24	0.07

Supplementary Table 1. Numbers of MC38 tumor-infiltrating immune cells per mg tumor for the experiment shown in Fig. 4A. Data are represented as mean +/- SEM; unpaired t-test.

Cytokine	NLK$^{+/+}$	NLK$^{fl/fl}$	p
IFNγ^{+} of CD8^{+} (%)	22.5 +/- 3.0	9.2 +/- 1.1	< 0.001
TNFα^{+} of CD8^{+} (%)	10.0 +/- 1.7	4.4 +/- 0.6	< 0.01
IL-2^{+} of CD4^{+} (%)	4.9 +/- 1.4	1.4 +/- 0.4	< 0.05
IFNγ^{+} of CD4^{+} (%)	2.8 +/- 0.5	1.4 +/- 0.2	< 0.05
TNFα^{+} of CD4^{+} (%)	11.2 +/- 2.0	4.1 +/- 0.8	< 0.01

Supplementary Table 2. Fractions of MC38 tumor-infiltrating lymphocytes expressing the indicated cytokines, related to the experiment shown in Fig. 4C. Data are represented as mean +/- SEM; unpaired t-test.

A

Surface marker	$NLK^{+/+}$	$NLK^{fl/fl}$	p
$PD\text{-}1^+$ of $CD8^+$ (%)	61.6 +/- 2.8	76.6 +/- 2.3	< 0.01
$TIM\text{-}3^+$ of $CD8^+$ (%)	43.2 +/- 2.7	60.0 +/- 2.5	< 0.001
$LAG\text{-}3^+$ of $CD8^+$ (%)	44.2 +/- 3.5	61.6 +/- 1.9	< 0.001
$CD39^+$ of $CD8^+$ (%)	47.3 +/- 2.5	62.6 +/- 2.9	< 0.01

B

Surface marker	$NLK^{+/+}$	$NLK^{fl/fl}$	p
PD-1	33,809 +/- 2,573	45,480 +/- 1,146	< 0.001
TIM-3	11,239 +/- 1,040	16,027 +/- 1,205	< 0.05
LAG-3	2,741 +/- 226	3,748 +/- 116	< 0.01
CD39	37,054 +/- 2,361	50,921 +/- 2,584	< 0.01

Supplementary Table 3. Surface marker expression of $CD8^+$ MC38 tumor-infiltrating lymphocytes. (A) Fractions of $CD8^+$ tumor-infiltrating lymphocytes expressing the indicated surface markers. **(B)** MFI of the indicated surface markers on $CD8^+$ tumor-infiltrating lymphocytes. Tables relate to the experiment shown in Fig. 5A. Data are represented as mean +/- SEM; unpaired t-test.
